# Colossal Thermal Expansion and Continuous Rolling Locomotion of a Robust Flexible Molecular Single Crystal

**DOI:** 10.1021/jacs.5c02006

**Published:** 2025-06-18

**Authors:** Bo Jing, Wenjie Kuang, Jinhe Li, Yongsheng Zhang, Songgu Wu, Liang Li, Panče Naumov, Junbo Gong

**Affiliations:** † School of Chemical Engineering and Technology, State Key Laboratory of Chemical Engineering, 12605 Tianjin University, Tianjin 300072, China; ‡ Haihe Laboratory of Sustainable Chemical Transformations, Tianjin 300192, China; § Smart Materials Lab, 167632 New York University Abu Dhabi, PO Box 129188, Abu Dhabi 129188, UAE; ∥ SAFIR Novel Materials Development Lab, Sorbonne University Abu Dhabi, PO Box 38044, Abu Dhabi 38044, UAE; ⊥ Center for Smart Engineering Materials, New York University Abu Dhabi, PO Box 129188, Abu Dhabi 129188, UAE; # Research Center for Environment and Materials, Macedonian Academy of Sciences and Arts, Bul. Krste Misirkov 2, Skopje MK-1000, Macedonia; ∇ Molecular Design Institute, Department of Chemistry, New York University, 100 Washington Square East, New York, NY 10003, United States

## Abstract

Amplifying microscopic or molecular perturbations to induce macroscopic mechanical effects in well-ordered molecular crystals is the foundation of the newly recognized potential of organic crystalline smart materials for soft organic electronics, optics, actuators, switches, and robots. Diverse molecular crystal actuators that transform external energy into mechanical motions have been prepared in the past decade, yet their spatiotemporal operational capability, adaptability, reversibility, and durability have not been fully explored. In this study, we present adaptive molecular single crystals that can respond to force, heat, and light, demonstrating mechanical flexibility, reversible expansion, and complex movements, including rolling and climbing locomotion. These crystals exhibit significant anisotropic thermal expansion within the temperature range from 303 to 413 K, expanding by approximately 4.8% along their longest axis. The exceptional flexibility and thermal expandability of this material enable quick and sustained locomotion of the single crystals when exposed to ultraviolet light. Our findings highlight the considerable yet underexplored potential of adaptive organic crystals that can be used as lightweight thermomechanical and photomechanical actuators.

## Graphical abstract

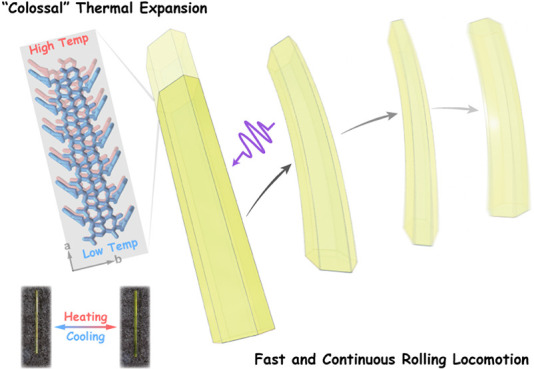

## Introduction

Living organisms have evolved and perfected the ability to convert chemical energy into mechanical work to respond dynamically to environmental stimuli.^1,2^ Inspired by nature, artificially manufactured actuators that range in complexity from simple matter to complex device architectures have been designed to demonstrate diverse biomimetic mechanical motions, such as bending,^3^ jumping,^4^ walking,^5,6^ crawling,^7^ and morphing,^8^ by stimulating changes in their structures. Unlike macroscopic actuators that have been refined over decades of engineering, molecular (primarily organic) crystals were proposed only recently as dynamic architectures. These materials have the potential to become a revolutionary platform for creating advanced actuators for several reasons. First, organic crystals commonly have intermediate Young’s moduli (1–25 GPa) and hardness (0.04–1.67 GPa), as established by global materials analysis of their mechanical properties,^9^ thereby being conveniently positioned between compact, rigid materials and lightweight, very soft and compliant materials. The combination of the two merits—pronounced softness and high robustness—makes organic crystals fulfill the prerequisites for being the material of choice in lightweight, soft micro- and macrodevices.^10–13^ Second, the densely arranged and long-range-ordered architectures generated by the self-assembly process are distinct from the structures of amorphous materials, enabling high-efficiency energy transduction and unique kinematic capabilities.^14,15^ Third, over the past decade, solid-state chemists and crystallographers have reported a wide variety of molecular crystals that demonstrate macroscopic dynamic effects, such as deformation, movement, or disintegration in response to external stimuli.^16,17^ In addition, certain crystals have been observed to exhibit the ability to self-heal autonomously after being damaged.^18–21^ The phenomena documented with these materials, referred to as “adaptive" or "dynamic" crystals, are not just serendipitous occurrences; they are actually now known to be quite abundant.^22^ In parallel with the reports of new dynamic crystals, attempts have been made to establish whether the mechanical effects can be “tuned” by either modifications of the molecules or by guiding the crystal packing, although the general principles are yet to be established.^23–28^ Important aspects such as response time, frequency of operation, cyclability and, especially, controllability, are yet to be explored.^29^ With this in mind, *restorative* molecular crystals for cyclic operations are often prioritized over the *disintegrative* ones, which are subject to stochastic events or motions in both space and time.^22,30^ Reversible molecule-level motions induced by isomerization, phase transition, and cyclodimerization reactions are well-known to lead to macroscopically recoverable deformation of bulk materials;^31–33^ candidates that meet this criterion hold the potential to be utilized as practical actuators. However, precise control over the deformation or motion of single crystals in a nondestructive manner remains a challenge.^34^ Herein, we report an example of a mechanically compliant crystal that exhibits continuous locomotion. The single crystals of this material can be deformed elastically or plastically upon the application of force on distinct crystal faces. Such flexible crystals undergo anomalous macroscopic expansion upon heating due to collective molecular motion within their spiral-like columnar structure. The adaptability and thermal expansion-related peculiarities of the single crystal serve to induce fast and perpetual locomotion driven by ultraviolet (UV) light. The durability of the thermal expansion and the cyclability of the rolling motion were validated by repeated stimulation.

## Results and Discussion

The focus of our interest was a proton-transfer compound, the Schiff base (*S*,*E*)-4-nitro-2-(1-((1,2,3,4-tetrahydronaphthalen-1-yl)­iminio)­ethyl)­phenolate, hereafter denoted NTIEP. Since in this work we do not address properties related to chirality, only the *S* enantiomer of the material, (*S*)-NTIEP, was synthesized by condensation between (*S*)-1,2,3,4-tetrahydronaphthalen-1-amine and 1-(2-hydroxy-5-nitrophenyl)­ethan-1-one (Figures 1a, S1 and S2) and studied. The absorbance spectra of (*S*)-NTIEP in solvents of different polarities revealed that proton transfer can occur in solution, and the equilibrium between the two tautomers shifts depending on the solvent polarity (Figure S3).^35^ Moreover, the fluorescence spectra showed a large Stokes shift in solution (Δλ = 193 nm, *n*-hexane), a result that is attributed to an excited-state intramolecular proton transfer (ESIPT). To complement the spectroscopic characterization, we performed time-dependent density functional theory calculations to evaluate the feasibility of ESIPT by determining the Gibbs free energy of the enol and keto forms in the excited state (the details are available in Figure S22). The results revealed a barrierless proton transfer pathway between the enol and keto forms in the excited state, indicating that (*S*)-NTIEP can undergo ESIPT in solution.^36^ Structure determination of (*S*)-NTIEP by X-ray diffraction (XRD) analysis showed that in the solid state the molecule exists as the NH tautomer (Figure 1b and Table S1).^37^ The relevant bond lengths determined by XRD analysis confirmed that the molecule is in its zwitterionic canonical form (Figure S4a).^38^ The crystal is in the orthorhombic system, space group *P*2_1_2_1_2_1_, and contains one molecule in the asymmetric unit that adopts a twisted conformation with a dihedral angle of 81.9° between the benzene ring and the aromatic ring of the tetrahydronaphthalene moiety (Figure S4b). The molecules form intramolecular N–H···O hydrogen bonds with an O···H distance of 1.747 Å.

**Figure 1:**
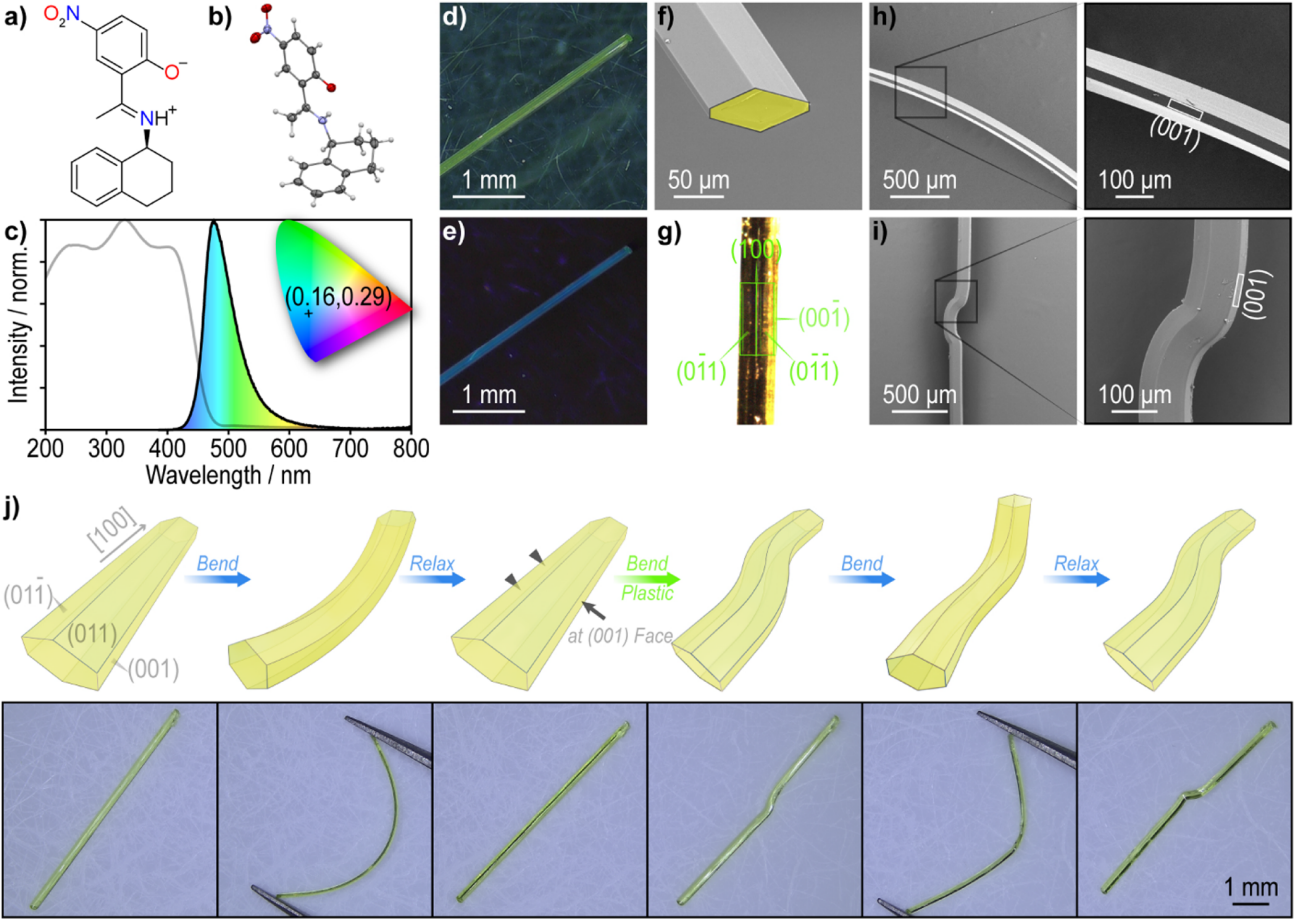
Structure, morphology, spectra, and mechanical flexibility of (*S*)-NTIEP crystals. (a) Chemical structure of (*S*)-NTIEP. (b) Molecular structure of (*S*)-NTIEP at 113 K represented as an ORTEP diagram (the ellipsoids are drawn at the 50% probability level). (c) Absorbance (gray line) and fluorescence (black line) spectra and a CIE 1931 chromaticity diagram (inset) with a CIE coordinate of (0.16, 0.29) showing the color of emission of the (*S*)-NTIEP crystals. (d, e) Microphotographs of a single crystal under bright (standard white light source, d) and dark (365 nm UV light source, e) fields. (f) Scanning electron microscopic (SEM) image of a naturally grown crystal with a false-colored cross-section highlighted for ease of inspection. (g) Indexing of the main facets based on single crystal XRD data. (h, i) SEM images of elastically (h) and plastically (i) bent crystals, with zoomed-in views of the bent regions shown on the right side. (j) Anisotropy in mechanical flexibility of a single crystal, illustrated with both schematic models and actual optical microphotographs.

Acicular crystals, typically 5–20 mm in length and 10–200 μm in width and thickness, were prepared by slow evaporation of an ethanol solution at ambient conditions (Figures S5 and S6). The crystals exhibit a faint blue emission upon 365 nm irradiation (Figure 1c–e). Inspection by scanning electron microscopy showed three pairs of parallel facets, where two sets are of nearly equal width, while the third pair is wider (Figure 1f). The facets were indexed based on the XRD data (Figures 1g and S7). The single crystals proved to be distinctly flexible: their widest faces underwent reversible (elastic) bending when stress was applied to both ends using tweezers, whereas irreversible (plastic) bending occurred when force was applied locally and perpendicular to the (001) face in a three-point bending geometry (Figure 1h,i and Movie S1). The single crystals can be forced to bend into a U-shape, with a calculated approximate elastic strain of about 3% (Figure S8). The surface mechanical properties were quantified by nanoindentation on the (001) face (Figure S9). The Young’s modulus and hardness were found to be 7.07 ± 0.22 and 0.22 ± 0.01 GPa, respectively. Many organic crystals with dual (elastic and plastic) deformation have been reported;^39,40^ consistent with other reports, the crystals of (*S*)-NTIEP showed to be initially elastic. However, as the elastic regime is quite narrow, the bending quickly becomes irreversible, the deformed crystals retain their bent shape, and thus, they can be considered plastic (Figure 1j). Some of the structural features that can account for the elastic or plastic bending of mechanically compliant crystals have been identified, and mechanisms for the observed bending have been advanced based on results from microfocus XRD diffraction.^41–44^ Since such analysis was unfortunately technically unaffordable to us, we analyzed the intermolecular interactions in (*S*)-NTIEP to be able to understand its mechanical properties (the details are available in Figure S23). During elastic bending, we anticipate that the distances between molecules in the outer arc will increase, while those in the inner arc will decrease, with the molecular rearrangement being facilitated by weak intermolecular interactions. When a crystal is plastically deformed upon application of stress on the (001) face, slippage of the molecular layers is likely to occur. This assumption is supported by the observation of surface features that indicate split layers in a crystal that has been subjected to excessive force (Figure S10).

In addition to the stress-induced deformation, some Schiff bases with higher conformational freedom are prone to rapid thermal responses.^45,46^ In order to check for thermally induced mechanical effects, microphotographs of an (*S*)-NTIEP single crystal were recorded from 303 to 413 K under a microscope equipped with a temperature-controlled stage (Figures 2a,b and S11a), up to about 10 K below its melting temperature (Figures S12 and S13). During heating, we observed that the single crystal underwent an exceptionally large expansion of 4.8% at 413 K along the [100] direction, while it also became more reflective and, hence, appeared slightly brighter (Figure S14). This color change is different from the distinct and readily detectable color change of thermochromic *N*-salicylideneanilines.^47^ Upon cooling, the thermally expanded single crystal gradually contracted to its original size, demonstrating reversibility of the thermally induced expansion.

**Figure 2:**
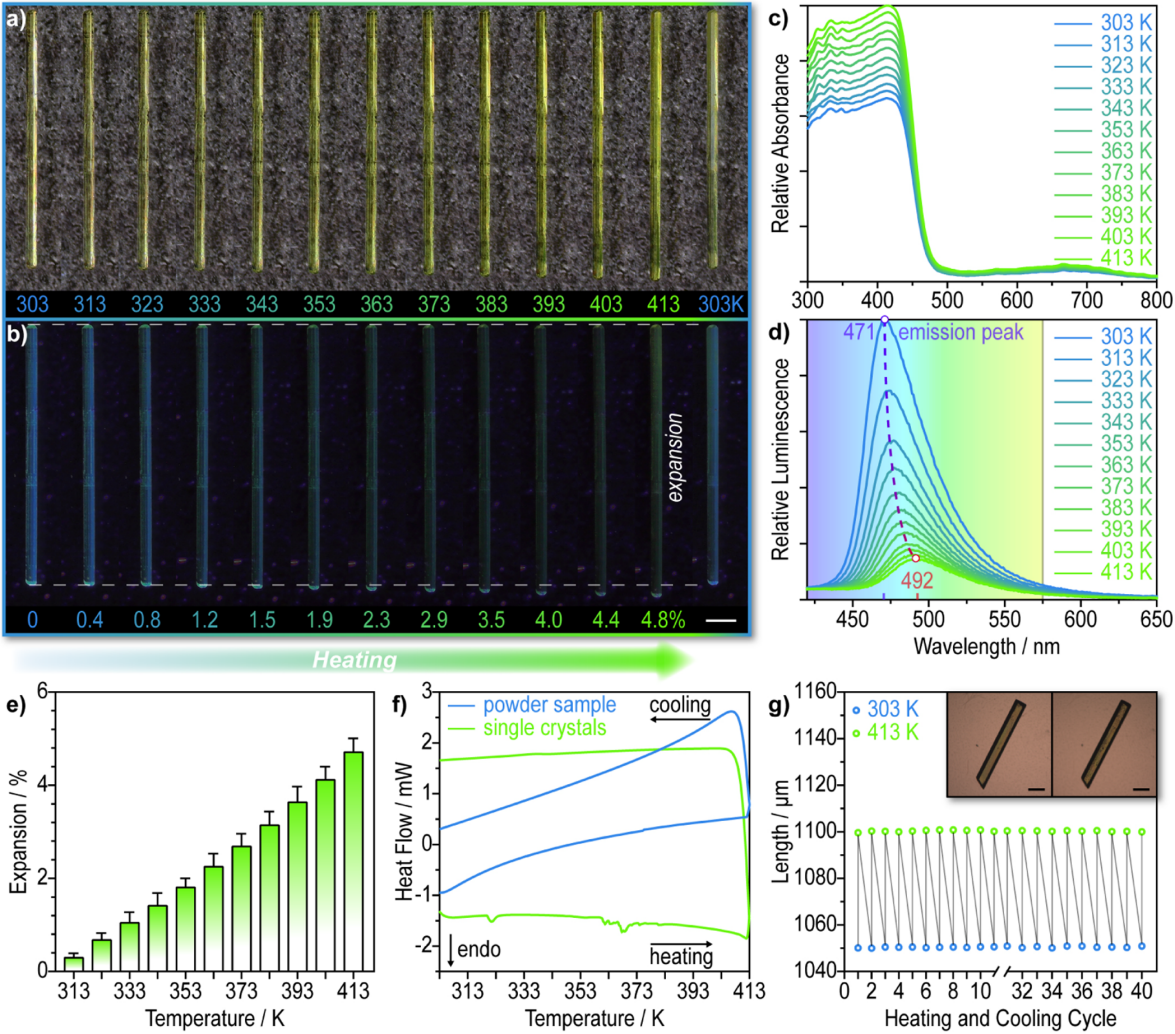
Reversible thermal expansion of heated (*S*)-NTIEP crystals. (a, b) Microphotographs of a single crystal during a heating–cooling cycle at an average rate of 2 K min^–1^ under white light (a) and 365 nm UV light (b). Scale bar: 500 μm. (c, d) Variable-temperature absorbance spectra (c) and fluorescence spectra (d) of the crystalline powder. The wavelengths of the maxima at 303 and 413 K are shown in nm. (e) Linear correlation between the percent length expansion of the crystal and the temperature. The error bars are the experimental standard deviation. (f) Differential scanning calorimetry curves of powder samples and single crystals recorded by heating and subsequent cooling. The heating and cooling rate was 10 K min^–1^. The sawtooth profile of single crystals was detected within a broad temperature range, and it implies movement of the crystal. (g) The length variation of the same crystal during heating–cooling cycles. Inset: microphotographs showing the crystal appearance before (left) and after (right) the thermal cycling experiment. Scale bar: 200 μm.

To further study the thermal responsive properties, the temperature-dependent absorbance spectra and fluorescence spectra of the crystals were recorded (Figure 2c,d). With increase in temperature, the absorption of all peaks enhanced without evolution of additional absorption bands in the visible region, while the blue fluorescence of the crystals weakened and redshifted from 471 nm at 303 K to 492 nm at 413 K. These results, complemented with variable-temperature infrared spectroscopic analysis (Figure S15), indicate the absence of molecular isomerization or proton transfer during heating between these two temperatures. The observed changes in emission may arise from thermally induced excited-state structural relaxation coupled to an increased contribution from nonradiative transitions.^48^ Additionally, statistical analysis of over a dozen single crystals with lengths ranging from 1 to 20 mm consistently revealed linear expansion along their longest axis (Figure 2e), and the thermal expansion is independent of the crystal dimensions (Movies S2 and S3). In rare instances, observations of this expansion by optical microscopy incidentally captured that the heated crystal suddenly jumped out of view (Figure S11b and Movie S4). Such jumping behavior of organic single crystals, known as the thermosalient effect,^2,49–55^ is commonly activated by a fast phase transition characterized by rapid structural changes that drive motion, but it can also be induced by light (photosalient effect)^56–63^ and mechanical force (mechanosalient effect).^19,64^ Variable-temperature powder XRD analysis showed that specific diffraction peaks gradually shifted toward lower angles due to lattice thermal expansion, without clear evidence of a phase transformation (Figure S16). The variable-temperature Raman spectra recorded around the temperature of jumping were in agreement with the results from the variable-temperature XRD analysis (Figure S17). Effects resembling the thermosalient effect have been very rarely documented when there is no phase transition,^65,66^ and in this case we hypothesize that, in absence of a phase transition, the crystal motion may be due to anisotropic thermal expansion (note that the thermal effects seen on heating in Figure 2f were identified as effects due to the crystal motion instead of a phase transition).

Single crystals with controllable and reversible shape deformation can serve as switching elements in electrical circuits.^67^ However, organic crystals—being based on noncovalent interactions as the weakest bonds—are thought to suffer from abrasion and even destruction after limited number of cycles, posing limitations with practical applications. As shown in Figure 2g, testing of the durability of the switching crystal of (*S*)-NTIEP showed stability over 40 heating–cooling cycles. Although fine cracks appeared on the crystal surface, the crystal’s overall integrity remained intact. Furthermore, we monitored the thermal expansion behavior of plastically bent crystals and, despite the presence of deformation-induced defects, observed no significant alteration in thermal responsiveness up to 408 K (Figure S18 and Movie S5). Our results provide an additional proof of the operational robustness of these organic crystals in operations that require precisely controllable deformation, such as those used in switching electric circuits.^68^

To provide more direct evidence, we attempted to determine the effect of temperature on the crystal structure by variable-temperature single crystal XRD. Starting at 303 K, a series of XRD diffraction data at intervals of 10 K were collected up to 413 K with increasing temperature (Figure S19 and Table S2). As shown in Figure 3a, upon heating, the crystallographic *a* axis expands whereas the *b* and *c* axes contract, without abrupt changes that would indicate a phase transformation. The principal coefficients of thermal expansion, calculated in the range 303–413 K by using PASCal,^69^ returned values of −78, −69, and 465 × 10^–6^ K^–1^ for *X*1, *X*2, and *X*3, which coincide with the crystallographic axes *c*, *b,* and *a*, respectively (Figure 3b). The significantly large volumetric thermal expansion coefficient, α_V_ = 323 × 10^–6^ K^–1^, arises from a combination between the uniaxial positive thermal expansion and the biaxial negative thermal expansion. Importantly, the thermal expansion along the *a* axis of 465 × 10^–6^ K^–1^ exceeds largely the normal values of molecular solids (<20 × 10^–6^ K^–1^). It stands out among the reported organic crystals (Table S3), and can be characterized as “colossal”, in line with the terminology used in the related literature for such strong expansivities.^70,71^

**Figure 3:**
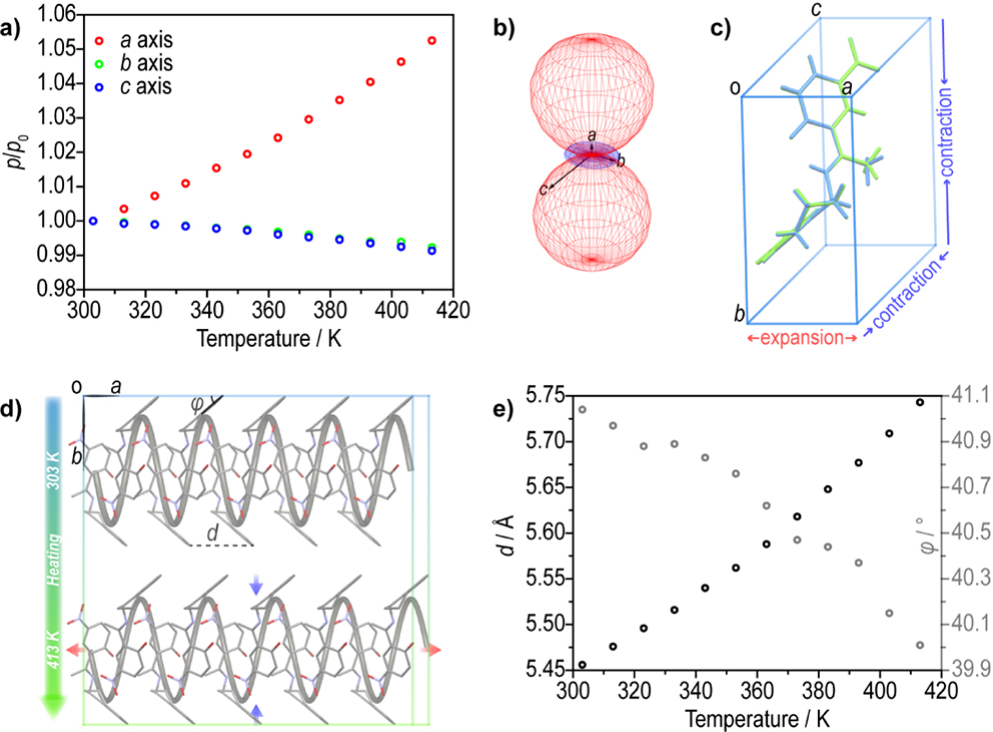
Structural changes in (*S*)-NTIEP crystal that occur with temperature. (a) Thermal variation of the unit cell axes from 303 to 413 K. (b) Plot of the expansivity indicatrix for (*S*)-NTIEP. The red and blue meshes represent positive and negative thermal expansion, respectively. (c) Overlapped unit cells at 303 K (blue) and 413 K (green), with the change in unit cell parameters that occur upon heating highlighted. (d) Schematic representation of the mechanism of thermal expansion. (e) Thermal variation in the distance (*d*, left ordinate, darker circles) between two adjacent molecules along the crystallographic *a* axis and the tilt angle (φ, right ordinate, lighter circles) of the aromatic ring of the tetrahydronaphthalene moiety relative to the [100] direction (illustrated in Figure 3d).

A set of 11 experimentally determined crystal structures (XRD) enabled us to visualize the structural changes that occur during the thermal expansion. Along the direction of anomalous positive thermal expansion, the (*S*)-NTIEP molecules are arranged in columns via weak C–H···O hydrogen bonds, C–H···C and C–H···π interactions, which dominate the primary growth direction of the needlelike crystals (Figure S23b). We carefully inspected and compared the changes in the structure between different temperature points. First, the molecular conformations were found to be nearly identical between the initial and final temperatures (Figure 3c). As the temperature was increased from 303 to 413 K, the distance (*d*) between two adjacent molecules parallel to the [100] direction increases, while the tilt angle (φ) of the aromatic ring of the tetrahydronaphthalene moiety relative to the [100] direction incrementally decreases (Figure 3e). This change resembles the stretching of a coil spring, which narrows in diameter as it is being stretched. In analogous action, in (*S*)-NTIEP the concomitant changes in *d* and φ cause expansion of the *a* axis concurrently with contraction of the *b* and *c* axes (Figure 3d).^72^ Based on a preliminary investigation of compounds analogous to (*S*)-NTIEP undergoing similar expansion (the details are available in Figure S24), we believe this colossal thermal expansion mechanism, arising from molecular rearrangement, could aid in establishing a strategy for screening and possibly engineering similar dynamic crystalline materials.

It has been well established that some salicylideneaniline crystals are photochromic due to proton transfer, combined with isomerization and twisting of their molecules, and the photochromism depends on the molecular conformation;^73,74^ nonplanar molecular conformations with sufficiently large twist angles are normally photochromic, while planar or nearly planar molecules are not. Inspired by the photomechanical bending of salicylideneaniline crystals reported by Koshima and the coworkers,^75,76^ we examined the behavior of single crystals of (*S*)-NTIEP upon exposure to UV radiation. A freshly prepared needle-shaped single crystal (8281 × 171 × 88 μm), with one of its ends fixed, was irradiated with a 365 nm UV light (approximately 400 mW cm^–2^). When the UV light was turned on and the crystal was irradiated in the [001] and [010] directions, it bent away from the light source, reaching a maximum angle of its tip displacement of 0.8° and 1.9°, respectively (Figure S20). Subsequently, when the UV radiation was terminated, the crystal instantly recovered its original shape in less than 0.1 s (Movie S6). This fast recovery is in contrast with the longer processes of shape recovery documented for some other photomechanical salicylideneaniline crystals upon illumination with visible light for several seconds.^75,76^ In addition, the X-ray crystallographic analysis of a single crystal after UV radiation showed no evidence of molecular photoisomerization. Thus, we speculate that the bending away from the light source could be attributed to expansion of the irradiated surface (Figure S20f), induced by the photothermal effect, which is likely amplified in materials having large thermal expansion coefficients.^77^ With spatially nonuniform UV light, we noticed sudden rolling of freely movable single crystals. This photoinduced rolling of the crystal was observed and recorded under a microscope during exposure to radiation from a fixed UV light source. In Figure 4a, the initial position of the single crystal is marked with a dashed box, and the moment just before it changed was set as 0 s (*i*). As soon as the UV light was switched on, the straight crystal bent almost simultaneously (*ii*) and rolled rapidly toward the lighting direction (*iii*–*vi*) until it rolled out of the field of view, with an average rolling speed of approximately 9.20 mm s^–1^ (Movie S7). It should be noted that the rolling speed cannot be simply compared to that of other analogous materials, as it is influenced by a number of factors such as the crystal size, irradiance, and substrate, among others.^78^ Moreover, reversing the position of the light source relative to the crystal can provide control over the rolling direction. When alternately driven by UV radiation in opposite directions, the back-and-forth motion of a single crystal can endure at least one hundred times without damage (Figure S21). We additionally examined the motion of crystals of the *R* enantiomer, which exhibited rolling directions identical to those of the (*S*)-NTIEP crystals. We also noticed that the crystal acquires a sufficient amount of kinetic energy to “climb” a slope under UV light (Figure 4b and Movie S8). Specifically, a slender single crystal was positioned at the bottom of a self-made slope with a 15° inclination from glass slides (*i*). When exposed to UV radiation, the crystal ascended the slope over a distance of roughly 25 mm in 18 s (*ii–iv*). Despite the moving speed and continuity being reduced relative to motion on a flat surface, the complex locomotion, which is analogous to climbing is, to our knowledge, reported here for the first time for a molecular crystal.

**Figure 4:**
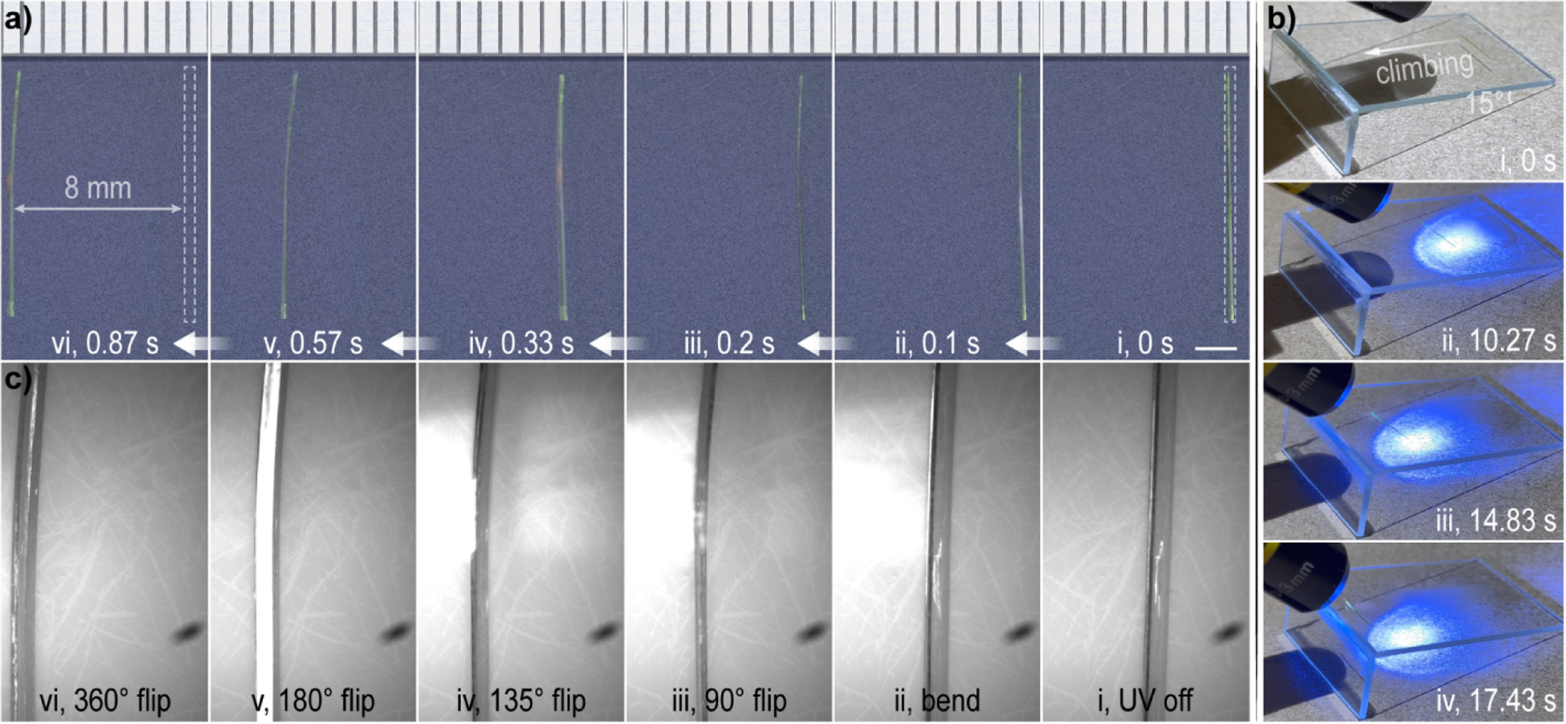
Modes of locomotion of (*S*)-NTIEP crystals. (a) Microphotographs of the rolling of a single crystal induced by exposure to UV radiation. Scale bar: 2 mm. (b) Snapshots showing “climbing” of a single crystal exposed to UV radiation. (c) Sequential snapshots of a single crystal undergoing a 360° roll under UV radiation, recorded with a high-speed camera.

To investigate the mechanism underlying this motility, we recorded the motion of crystals under UV radiation using a high-speed camera operating at 2000 frames per second. The motion of a well-defined single crystal was captured in a slow-motion video (Movie S9), and sequential stills extracted (Figure 4c) provided visualization of the kinematic details. Based on these observations, we hypothesized a possible mechanism for the continuous rolling locomotion. In Figure 5, surfaces A and B of the single crystal, which are mostly exposed to light, are highlighted. When exposed to UV light, surface A undergoes significant photothermal expansion, while surface B expands only slightly due to lower light absorption caused by the reflection. This differential expansion between surfaces A and B induces a nonuniform stress distribution within the crystal. This stress gradient, combined with the resultant bending deformation, could generate a torque around the contact point. Concurrently, the deformation shifts the center of mass forward, perturbing the mechanical equilibrium of the crystal. Under continuous UV radiation, the sustained expansion maintains the stress gradient, which is hypothesized to drive persistent torque generation, enabling the crystal to continuously roll toward the light source. We acknowledge that further quantitative analysis of torque dynamics and stress distribution would be required to validate this hypothesis and to resolve the local structural evolution in the irradiated regions.

**Figure 5:**
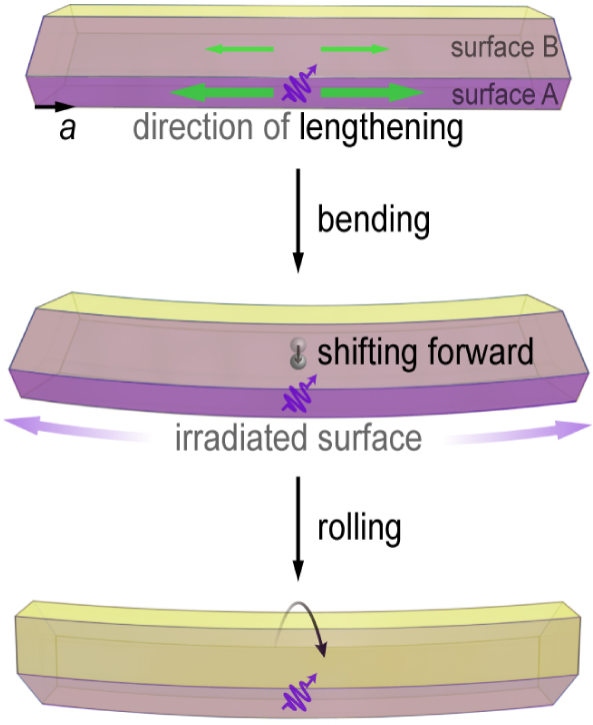
Schematic representation of the possible mechanism of locomotion of (*S*)-NTIEP crystals induced by exposure to UV radiation.

## Conclusions

Organic molecular crystals assembled via weak intermolecular forces are prone to damage, yet these finely tunable interactions also enable them to function as dynamic systems that can be manipulated by external stimuli. We present here a versatile and robust molecular crystal that exhibits anisotropic elasticity and plasticity, colossal thermal expansion, and rolling locomotion in response to force, heat, and light, respectively. First, the anomalous thermal expansion phenomenon was systematically investigated, the structural changes were visualized by determining a set of crystal structures at various temperatures, and the structural basis of the spring-like columnar arrangement was identified. Notably, the single crystal demonstrates reversible expansion and contraction over more than 40 cycles without decrease in performance. The durable and controllable shape deformation renders these and possibly other similar materials ideal candidates for microcircuit components, such as switches. Second, spatially nonuniform UV light causes slender single crystals to roll swiftly and continuously toward the light source, even ascending slopes. This mechanism allows molecular crystals to act as an efficient energy-conversion vehicle that transforms the input energy to mechanical work. The integration of multiple intriguing properties within a single crystal is rare, underscoring the appeal of organic crystals as smart crystalline materials. This work demonstrates that beyond simple deformation organic crystals possess remarkable adaptability and capability for complex motions. These assets are fundamental to the envisioned operations of these materials as soft actuators and other dynamic components.

## Methods

### Materials

1-(2-Hydroxy-5-nitrophenyl)­ethan-1-one (98%) was obtained from Tianjin Heowns Biochem Technologies LLC. (*S*)-1,2,3,4-Tetrahydronaphthalen-1-amine (98%) was obtained from Meryer (Shanghai) Biochemical Technology Co., Ltd. Ethanol (AR, water content ≤ 0.3%), *n*-hexane (97%), cyclohexane (99.5%), and acetonitrile (99%) were obtained from Shanghai Aladdin Biochemical Technology Co., Ltd. DMSO-*d*_6_ was purchased from Cambridge Isotope Laboratories. All chemicals were used without further purification.

### Characterization

The proton nuclear magnetic resonance (^1^H NMR) spectrum was recorded on Ascend 400 MHz spectrometer (Bruker), and the high-resolution mass spectrum (HRMS) was recorded by using a Q Exactive Plus spectrometer (Thermo Scientific). The absorption spectra in solution were recorded on a Cary 60 UV–vis spectrophotometer (Agilent), the absorption spectra of crystals were recorded on a UV-3600 spectrophotometer (Shimadzu), and the fluorescence spectra were recorded on an FLS1000 spectrometer (Edinburgh Instruments). The infrared spectra were recorded on a VERTEX 80v (Bruker) in a nitrogen atmosphere. The powder X-ray diffraction data were collected by using a MiniFlex600 diffractometer (Rigaku), and the variable-temperature X-ray diffraction data were collected by using a D8 Discover diffractometer (Bruker). The Raman spectra were recorded by using a LabRAM HR Evolution spectrometer (HORIBA) with an excitation wavelength of 785 nm. The nanoindentation tests were conducted with a G200 nanoindenter (KLA Instruments). The thermogravimetric analysis (TGA) was performed by using a TGA/DSC 1 STARe system (Mettler Toledo). The differential scanning calorimetry (DSC) analysis was performed on a DSC 1 STARe system (Mettler Toledo). A stereoscopic microscope SMZ745T (Nikon) equipped with a digital camera and a scanning electron microscope TM3000 (Hitachi) were used to record the micrographs of straight and bent crystals. The hot-stage microscopic observation was carried out by using a Linkam system, including a temperature-controlled stage LNP94/2 mounted on a microscope CKX53 (Olympus). The temperature was also controlled using an open temperature-controlled hot stage (WIGGENS) for experimental observation. A 365 nm monochromatic ultraviolet light with adjustable power emitted via LTWS-UV-D6 (Shenzhen LTWS Optoelectronic Technology) with a spot diameter of 3/10 mm was used as the UV light source.

### Single Crystal X-ray Diffraction

A suitable crystal was glued to a thin glass fiber, and the diffraction data were collected on an XtaLAB Synergy Custom system, HyPix diffractometer (Rigaku). The diffraction data were initially collected at 303 K, and then a series of data were collected at intervals of 10 K as the temperature was increased to 413 K, and finally collected when the temperature decreased to 303 K. The temperature of the crystal was controlled by a Cryostream 800 Cooler (Oxford Cryosystems). The structure was solved with the SHELXT structure solution program in Olex2 by using intrinsic phasing methods, and refined with the SHELXL refinement package by using least-squares methods.^79,80^ The crystallographic data are summarized in Table S2.

## Supplementary Material





















## Notes

Movie S1: Mechanical flexibility of a single crystal (MP4)
Movie S2: Thermal expansion of a single crystal (example 1) (MP4)
Movie S3: Thermal expansion of two single crystals (examples 2 and 3) (MP4)
Movie S4: Jumping behavior of a single crystal (MP4)
Movie S5: Thermal expansion of a plastically bent crystal (MP4)
Movie S6: Bending behavior of a single crystal, with one end fixed, upon exposure to UV radiation (MP4)
Movie S7: Rolling of a single crystal induced by exposure to UV radiation (MP4)
Movie S8: “Climbing” of a single crystal induced by exposure to UV radiation (MP4)
Movie S9: Rolling locomotion of a single crystal recorded with a high-speed camera (MP4)
Detailed experimental procedures, ^1^H NMR spectrum, absorption and fluorescence spectra in solution, powder X-ray diffraction pattern, face indexing, infrared spectra in the solid state, additional graphs and tables, methods used in the calculations, and discussion of the mechanical properties (PDF)
