# Supplementary material for: Colossal Thermal Expansion and Continuous Rolling Locomotion of a Robust Flexible Molecular Single Crystal
Source: J Am Chem Soc. 2025 Jun 18;147(26):22488–97. doi: 10.1021/jacs.5c02006 (PMC12232327; doi:10.1021/jacs.5c02006)
Supplement: Supplementary file 10 [file ja5c02006_si_010.pdf]

Supporting Information

Colossal Thermal Expansion and Continuous Rolling Locomotion of a Robust Flexible Molecular Single Crystal

Bo Jing,[1,#] Wenjie Kuang,[1,#] Jinhe Li,[1] Yongsheng Zhang,[1] Songgu Wu,[1,2]* Liang Li,[3,4] Panče Naumov[3,5,6,7]* and Junbo Gong[1,2]*

[1]*School of Chemical Engineering and Technology, State Key Laboratory of Chemical Engineering, Tianjin University, Tianjin 300072, China*

[2]*Haihe Laboratory of Sustainable Chemical Transformations, Tianjin 300192, China*

[3]*Smart Materials Lab, New York University Abu Dhabi, PO Box 129188, Abu Dhabi, UAE*

[4]*SAFIR Novel Materials Development Lab, Sorbonne University Abu Dhabi, PO Box 38044, Abu Dhabi, UAE*

[5]*Center for Smart Engineering Materials, New York University Abu Dhabi, PO Box 129188, Abu Dhabi, UAE*

[6]*Research Center for Environment and Materials, Macedonian Academy of Sciences and Arts, Bul. Krste Misirkov 2, MK-1000 Skopje, Macedonia*

[7]*Molecular Design Institute, Department of Chemistry, New York University, 100 Washington Square East, New York, NY 10003, USA*

[#]Equal contribution.

*Corresponding authors emails:

wusonggu@tju.edu.cn (S.W.), pance.naumov@nyu.edu (P.N.), junbo_gong@tju.edu.cn (J.G.)

Legends for the Supplementary Videos

Movie S1: Mechanical flexibility of a single crystal (MP4).

Movie S2: Thermal expansion of a single crystal (example 1) (MP4).

Movie S3: Thermal expansion of a single crystal (example 2) (MP4).

Movie S4: Jumping of a single crystal (MP4).

Movie S5: Thermal expansion of a plastically bent crystal (MP4).

Movie S6: Bending of a single crystal, with one end fixed, upon exposure to UV radiation (MP4).

Movie S7: Rolling of a single crystal induced by exposure to UV radiation (MP4).

Movie S8: “Climbing” of a single crystal induced by exposure to UV radiation (MP4).

Movies S9: Rolling of a single crystal recorded with a high-speed camera (MP4).

Supplementary Methods

1. **(*S*,*E*)-4-nitro-2-(1-((1,2,3,4-tetrahydronaphthalen-1-yl)iminio)ethyl)phenolate ((*S*)-NTIEP):** (*S*)-1,2,3,4-tetrahydronaphthalen-1-amine (736 mg, 5 mmol) and 1-(2-hydroxy-5-nitrophenyl)ethan-1-one (906 mg, 5 mmol) were added into 15 mL ethanol in a reaction bottle and the resulting mixture was refluxed with stirring for 6 hours. After cooling to room temperature, the reaction mixture was filtered, washed with ethanol and dried under vacuum. Further recrystallization from hot ethanol yielded yellow needle (*S*)-NTIEP crystals (1.26 g, 81.2%). **^{1}H NMR** (400 MHz, DMSO-d$_6$, Figure S1): δ = 17.39 (d, J = 7.2 Hz, 1H), 8.62 (d, J = 3.0 Hz, 1H), 8.05 (dd, J = 9.5, 3.0 Hz, 1H), 7.31-7.14 (m, 4H), 6.59 (d, J = 9.5 Hz, 1H), 5.42 (q, J = 6.7, 6.0 Hz, 1H), 2.90 (dt, J = 17.0, 6.2 Hz, 1H), 2.84 (s, 3H), 2.85-2.75 (m, 1H), 2.16 (td, J = 9.6, 9.0, 5.4 Hz, 1H), 1.96-1.83 (m, 3H). **HRMS** (ESI, Figure S2): Calcd for $C_{18}H_{18}N_2O_3$ [M]: 310.138, found: 311.138 $[M+H]^+$.

2. **Preparation of the centimeter-scale (*S*)-NTIEP single crystals:** A solution of (*S*)-NTIEP in ethanol (5 mg/mL) was prepared and placed in a Petri dish at room temperature. After solvent evaporated in 3-5 days, yellow-green needle-like centimeter-size crystals of (*S*)-NTIEP were obtained and characterized by using powder X-ray diffraction.

3. **Sample preparation for SEM characterization:** Acicular (*S*)-NTIEP single crystals were bent and fixed on the conductive tape manually by tweezers and a fine needle, and sputter-coated with gold before observation. SEM analysis was conducted at an accelerating voltage of 15 kV.

4. **Observation of the reversible thermal expansion behaviors:** Some as-prepared (*S*)-NTIEP single crystals with lengths ranging from micrometers to centimeters were selected, placed on a glass slide, heated in a temperature-controlled stage by using nitrogen gas from 303 K to 413 K, and observed under a microscope. To ensure the accuracy of the length measurement, images of the single crystal were recorded at each temperature point.

5. **Observation of the reversible bending under UV irradiation:** One end of a selected needle-like single crystal of (*S*)-NTIEP was affixed with clay, and its bending under UV radiation was observed in different directions under a microscope.

6. **Observation of the continuous rolling locomotion towards UV light:** A needle-like single crystal of (*S*)-NTIEP was placed on a glass plate, irradiated by a 365 nm monochromatic ultraviolet light with adjustable power, and observed under a microscope. The crystal motion was recorded as a video, and screenshots were extracted from the video recording.

7. **Observation of the climbing locomotion towards UV light:** A needle-like single crystal of (*S*)-NTIEP was placed on a self-made slope with an approximate tilt angle of 15°, irradiated with a 365 nm monochromatic ultraviolet light, and observed with a naked eye. A video of the process was also recorded, and screenshots were extracted from the video.

Supplementary Figures

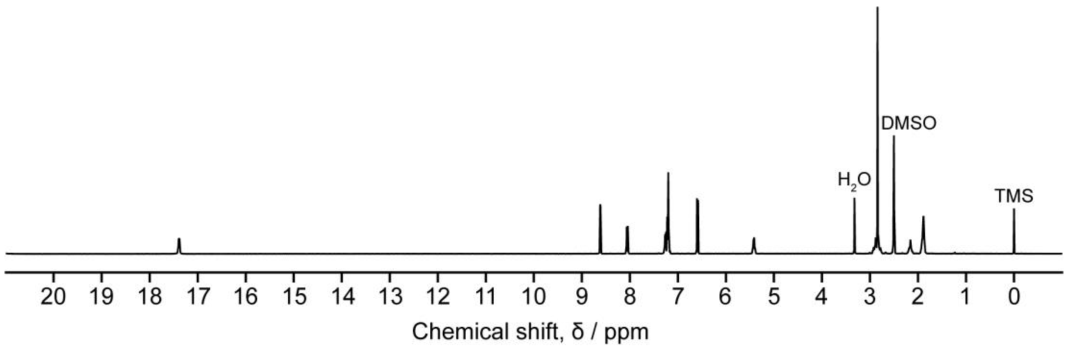

Figure S1. ^{1}H NMR spectrum of (*S*)-NTIEP in DMSO-d_6.

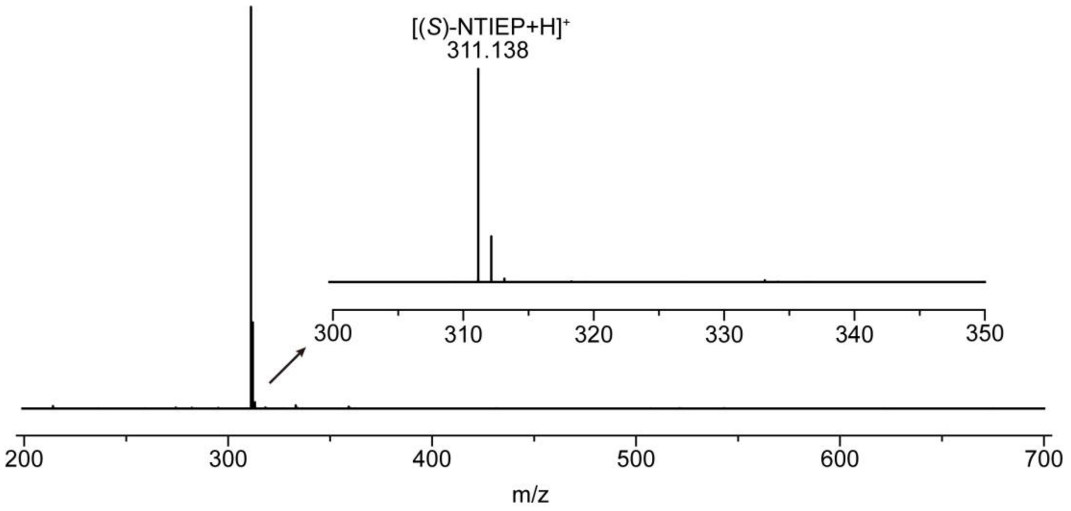

Figure S2. The HRMS of (*S*)-NTIEP.

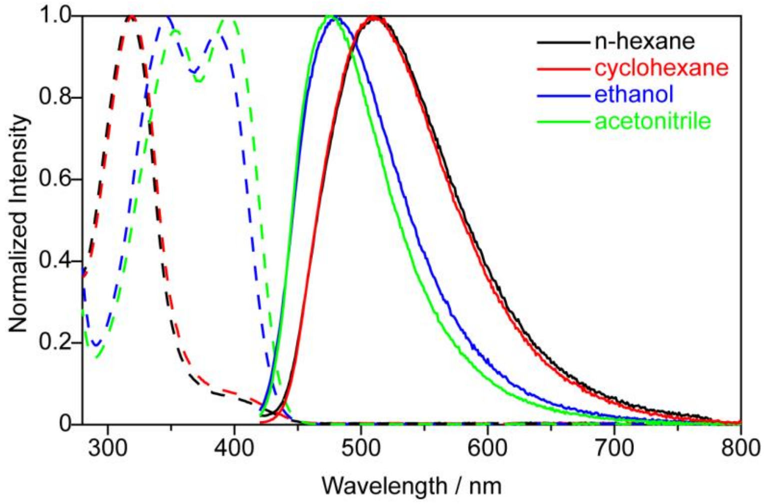

Figure S3. Absorbance spectra (dashed line) and fluorescence spectra (solid line) of (*S*)-NTIEP recorded in solvents of different polarities.

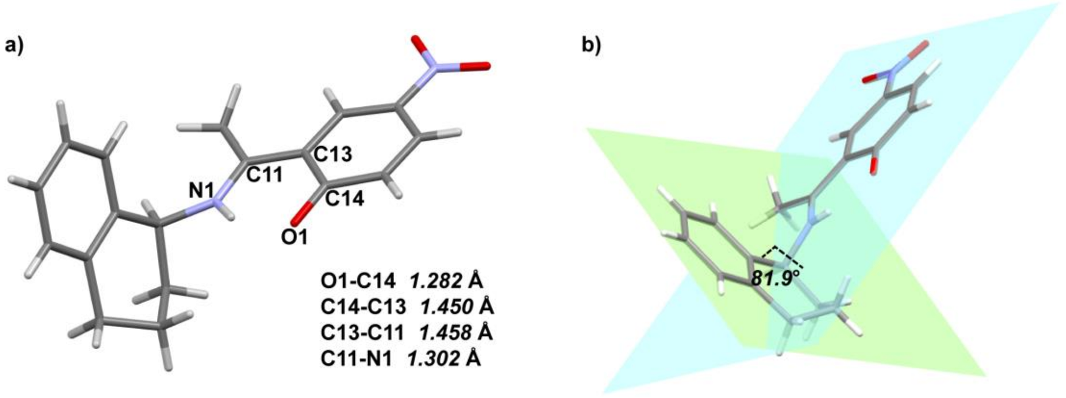

Figure S4. Selected bond lengths (a) and dihedral angle (b) in (*S*)-NTIEP.

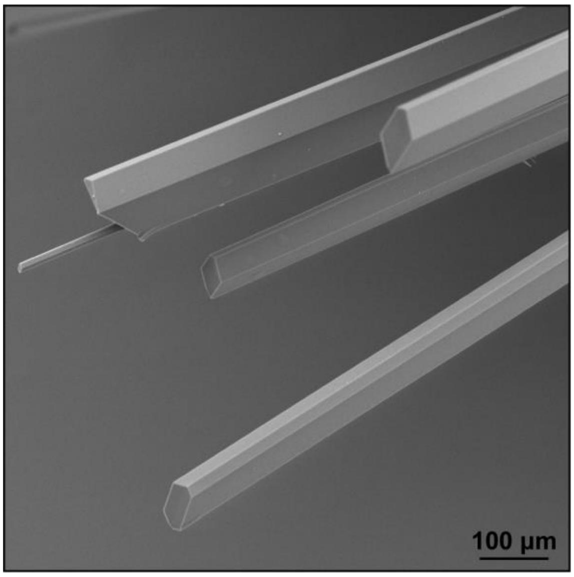

Figure S5. Scanning electron microscopic images of prepared (*S*)-NTIEP crystals.

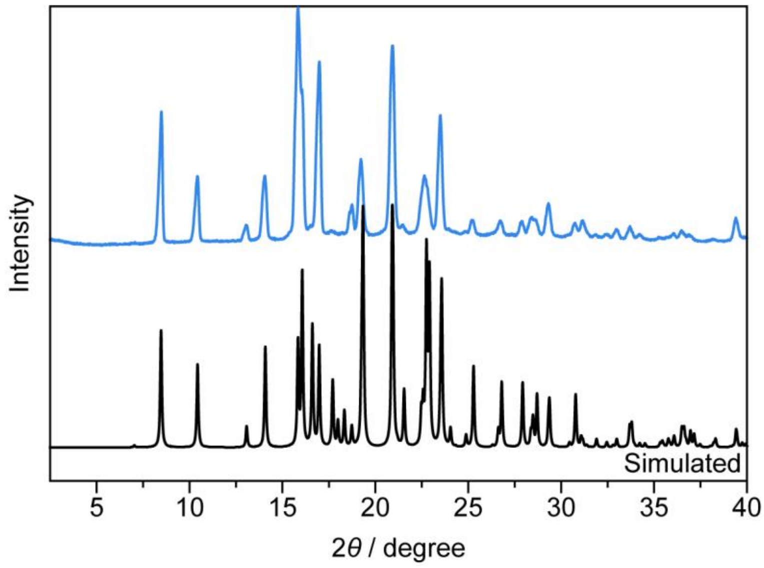

Figure S6. Simulated crystallographic diffraction pattern of (*S*)-NTIEP and powder X-ray diffraction pattern of the prepared (*S*)-NTIEP crystals.

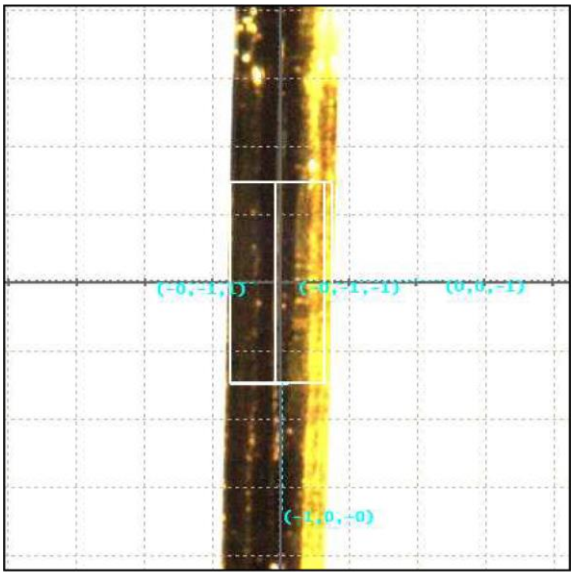

Figure S7. Face indexing of an (*S*)-NTIEP single crystal.

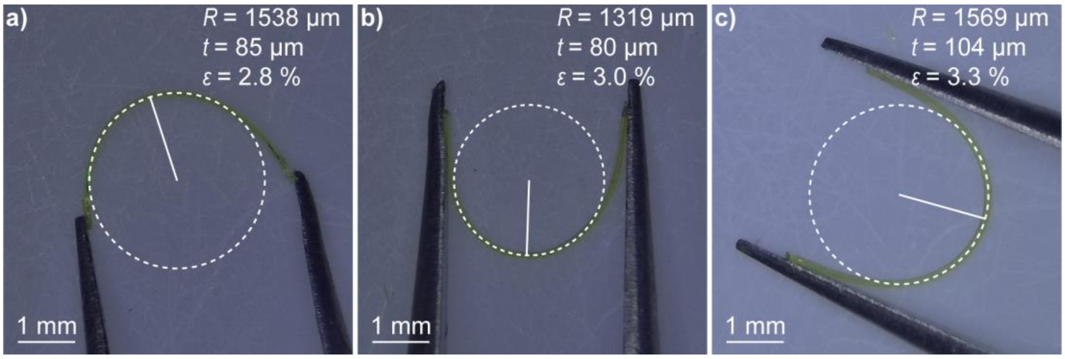

Figure S8. Elastic strain of single crystals of (*S*)-NTIEP estimated by using the Euler-Bernoulli equation: $\varepsilon = t/(2R)$, where t is the thickness and R is the approximate radius of the curved region.

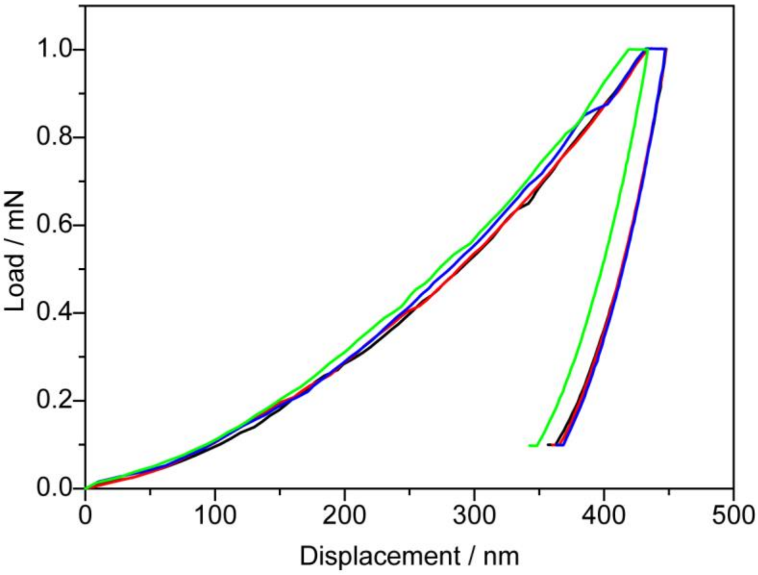

Figure S9. Load-depth curves obtained from nanoindentation tests on the (001) face.

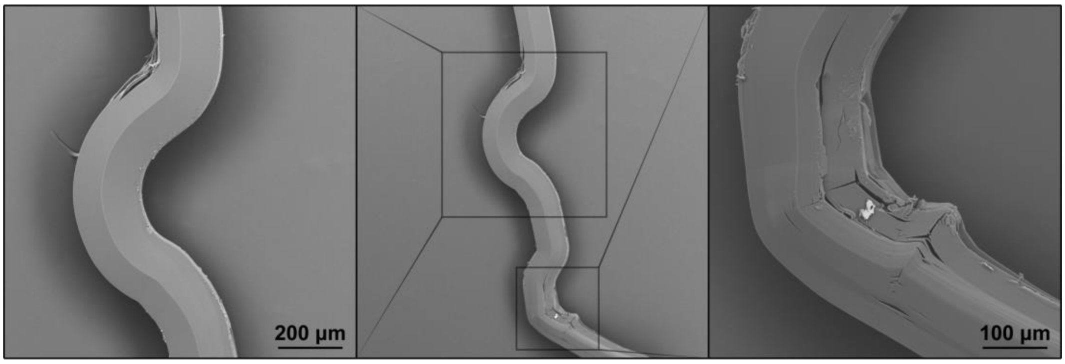

Figure S10. Scanning electron microscope images of a plastically bent (*S*)-NTIEP crystal, with the bent region zoomed in.

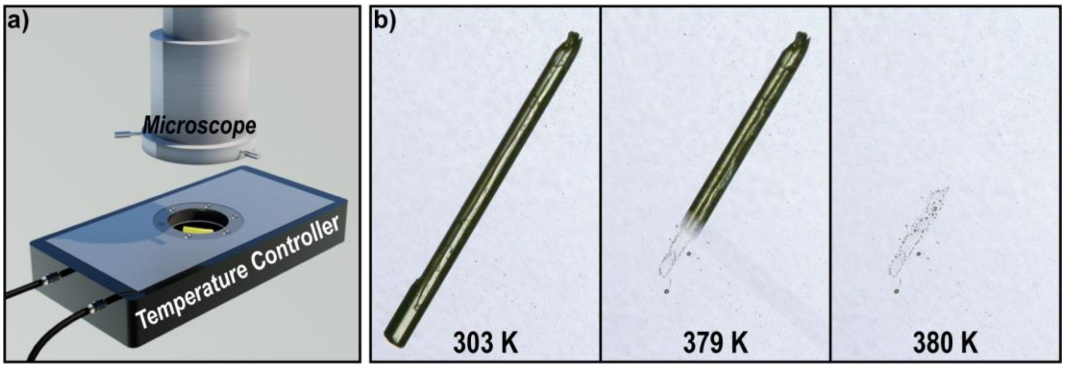

Figure S11. (a) Schematic representation of experimental device for observing thermal expansion behavior. (b) Microphotographs of the jumping behavior of (*S*)-NTIEP single crystal heated from 303 K to 380 K.

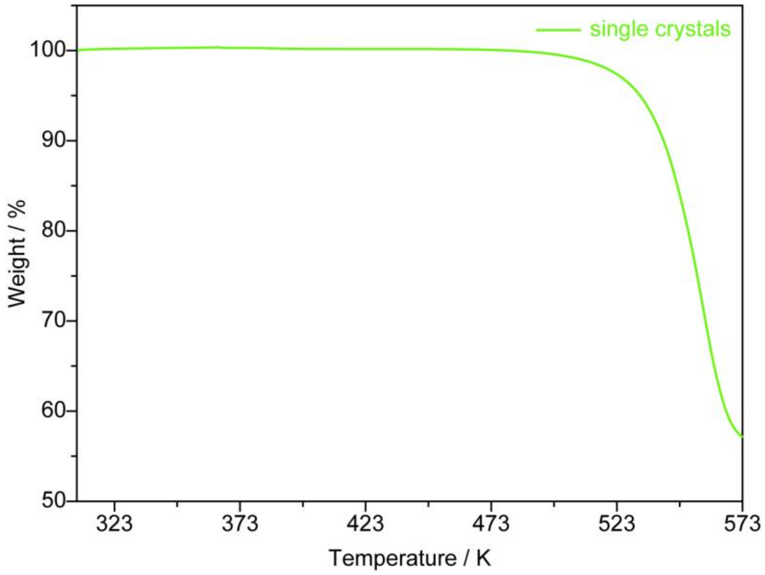

Figure S12. TGA curve of single crystals of (*S*)-NTIEP.

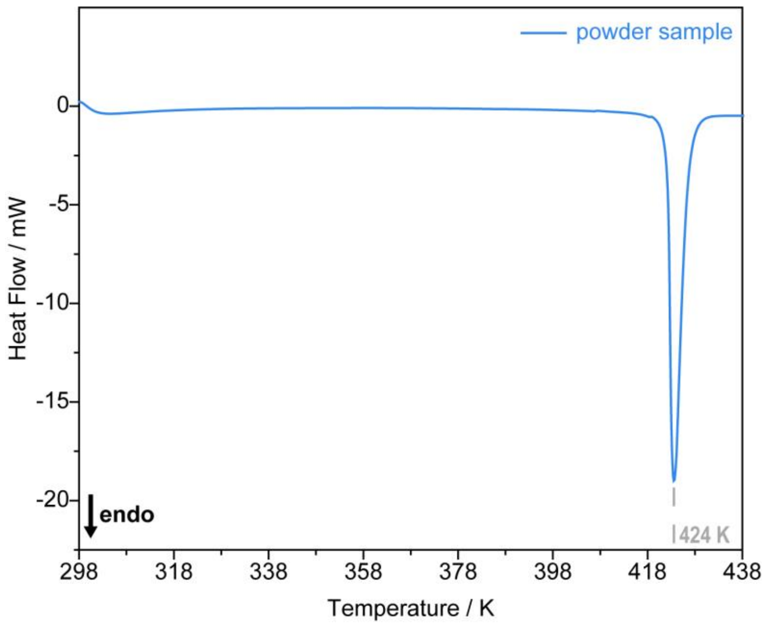

Figure S13. DSC curve of powder sample of (*S*)-NTIEP.

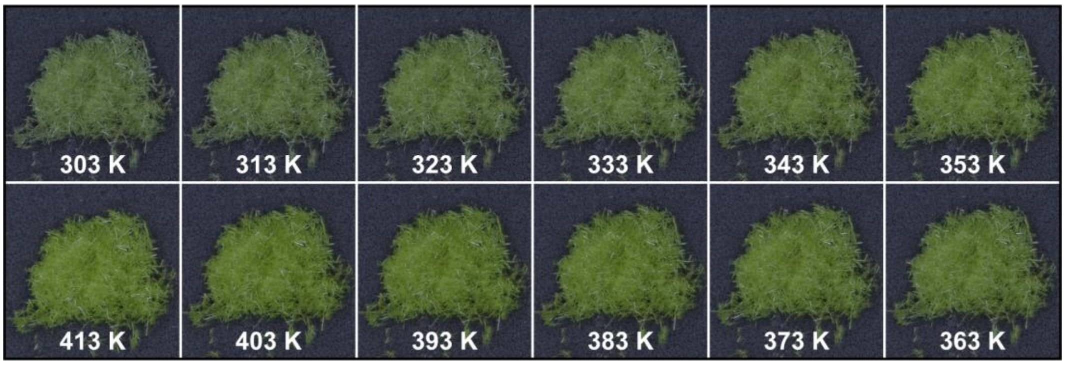

Figure S14. Microphotographs of (*S*)-NTIEP powder during the heating process under bright (standard white light source) field.

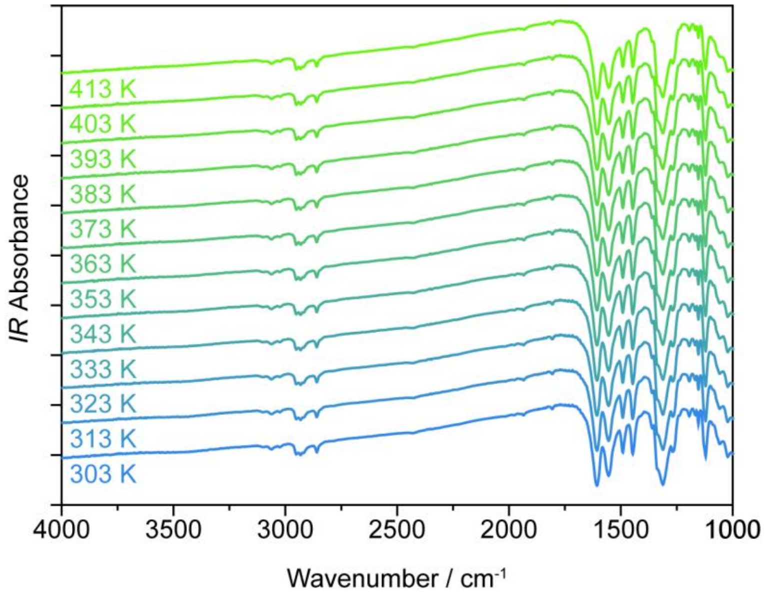

Figure S15. Variable-temperature infrared spectra of (*S*)-NTIEP crystals.

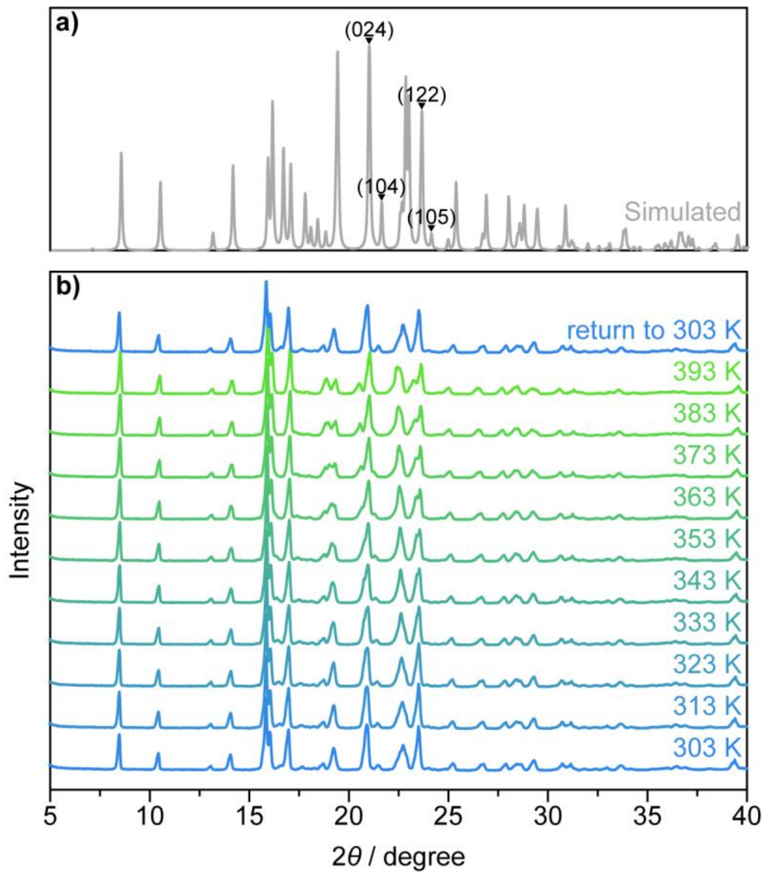

Figure S16. (a) Simulated crystallographic diffraction pattern (from the single crystal structure at 303 K) and (b) variable-temperature powder X-ray diffraction pattern of (*S*)-NTIEP crystals.

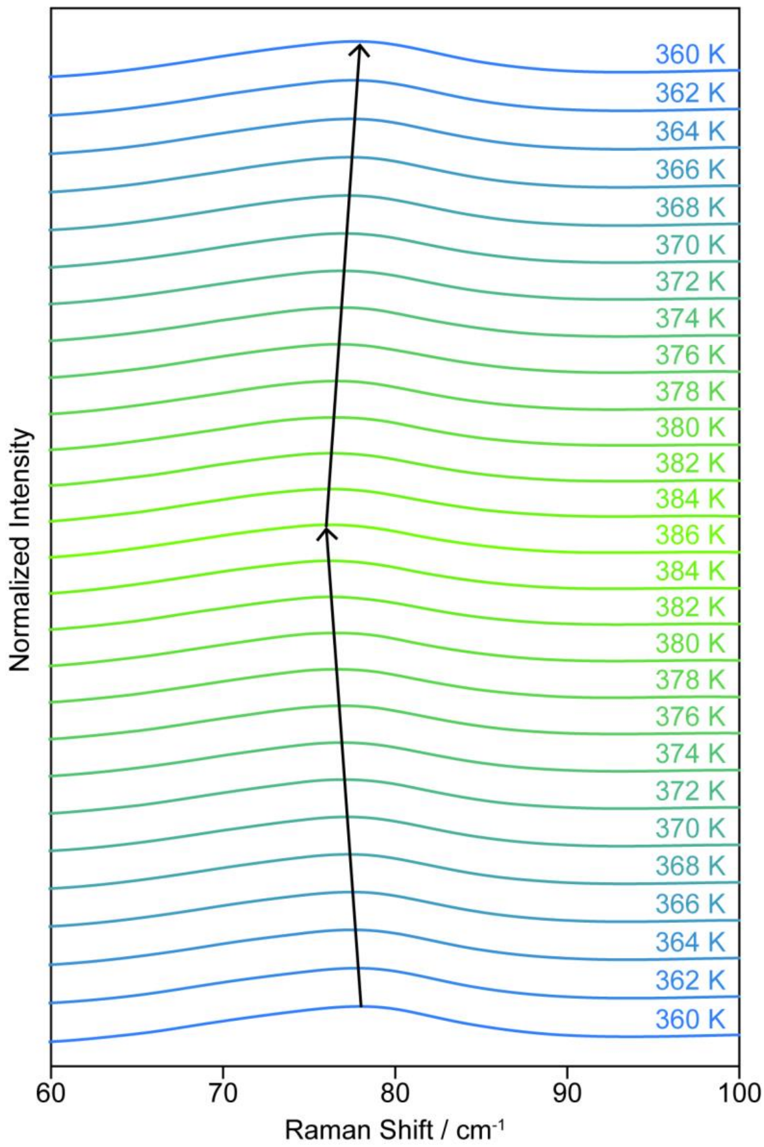

Figure S17. The 60 – 100 cm^{-1} region in the variable-temperature Raman spectrum of (*S*)-NTIEP single crystal recorded at 2 K intervals from 360 K to 386 K during a heating-cooling cycle, showing a continuous and hysteresis-free frequency shift due to thermal expansion.

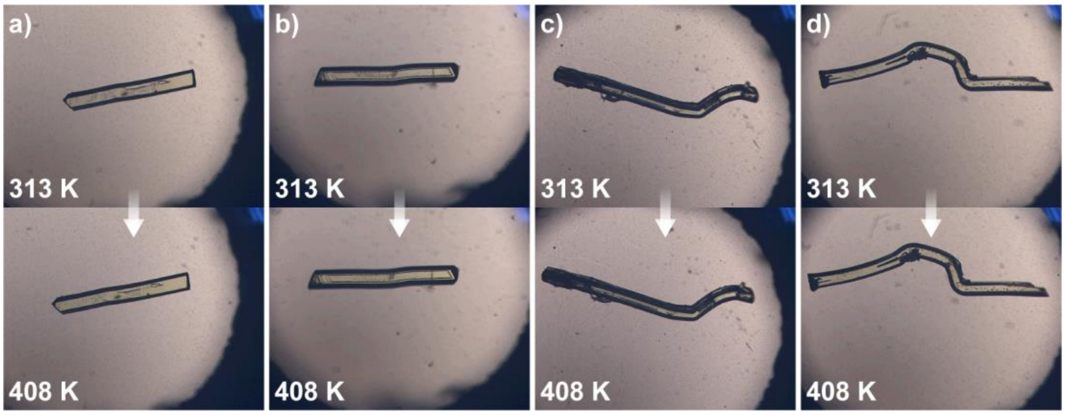

Figure S18. Microphotographs of plastically bent (*S*)-NTIEP single crystals heated from 313 K to 408 K.

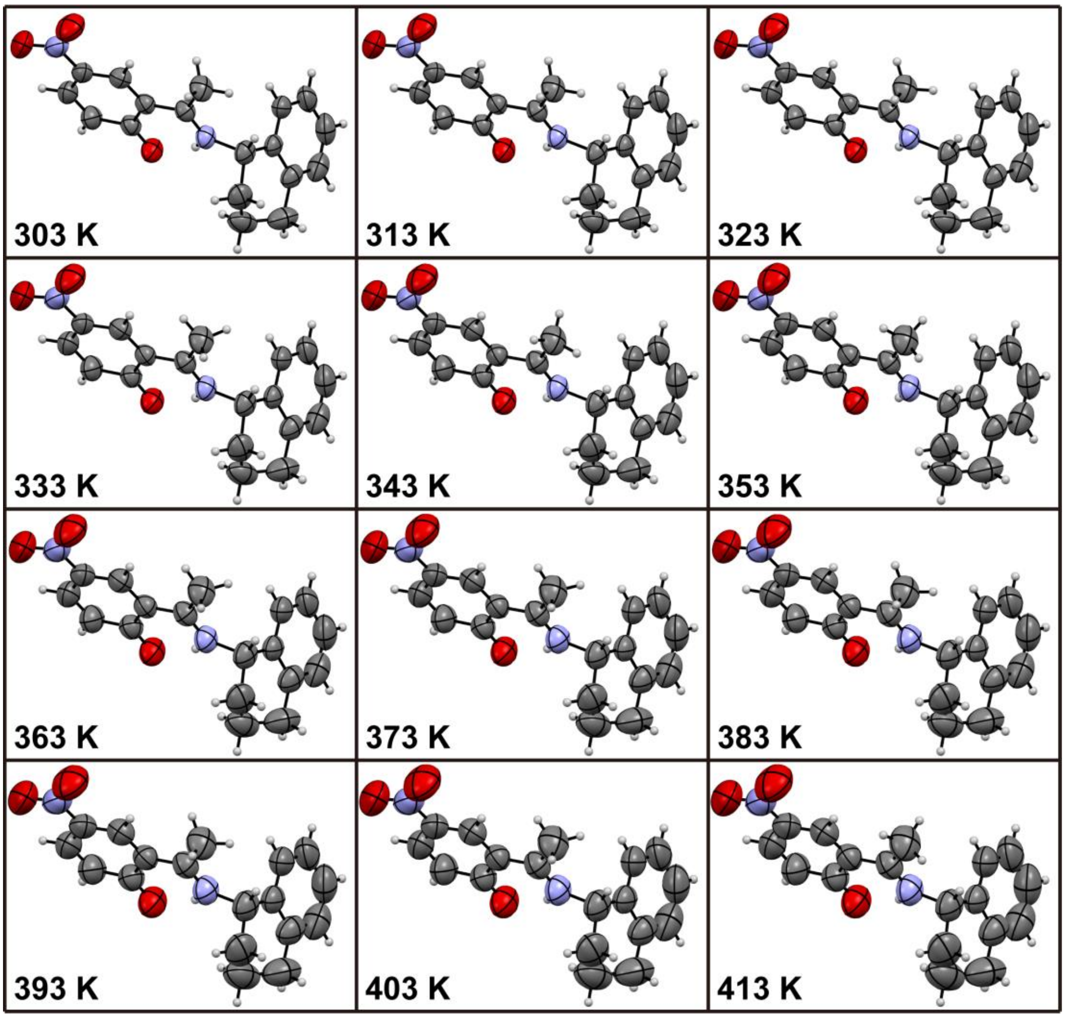

Figure S19. Thermal ellipsoid plots of (*S*)-NTIEP at different temperatures (C: black, N: purple, O: red, and H: white). The ellipsoids are drawn at the 50% probability level.

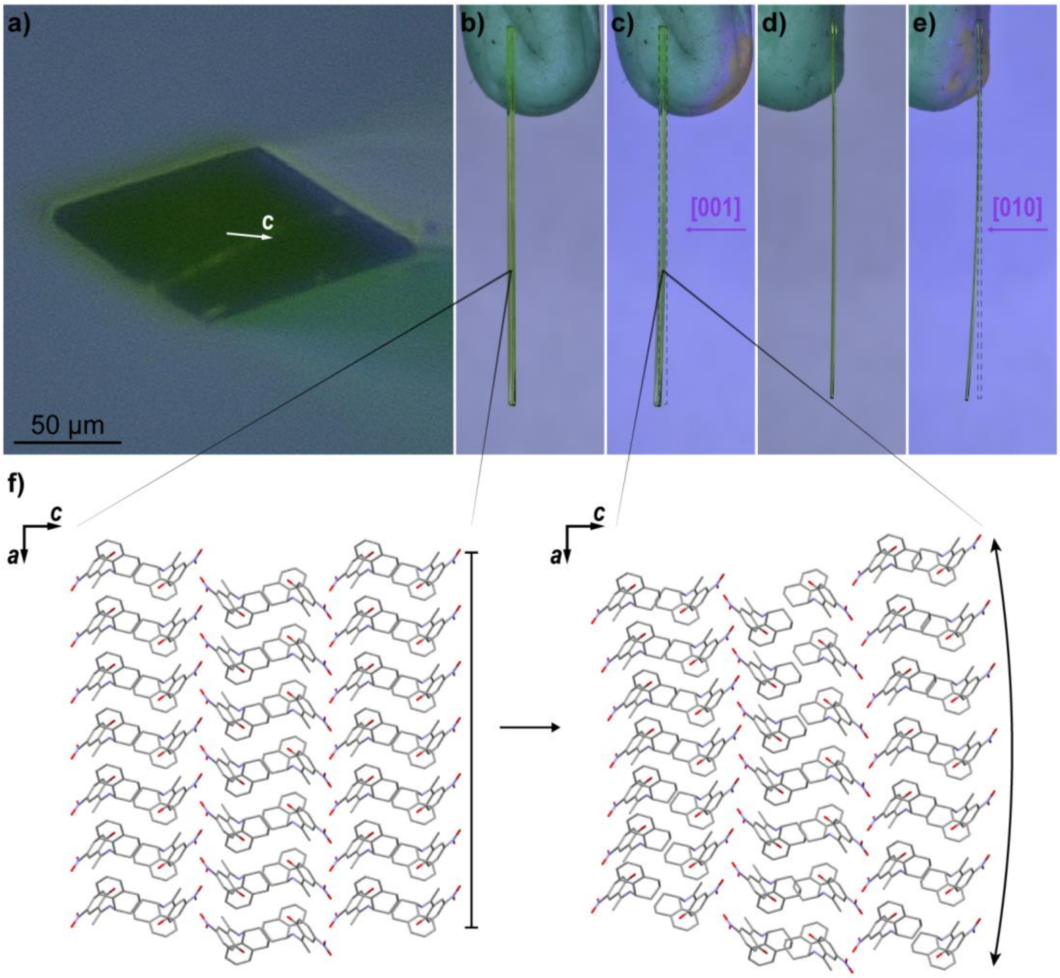

Figure S20. Microphotographs of the (*S*)-NTIEP single crystal (a) at cross-section face, and (b, d) before and (c, e) during UV irradiation along [001] and [010] directions. The initial position of the crystal before UV radiation is marked with a dashed line. (f) Schematic illustration of the bending upon UV irradiation.

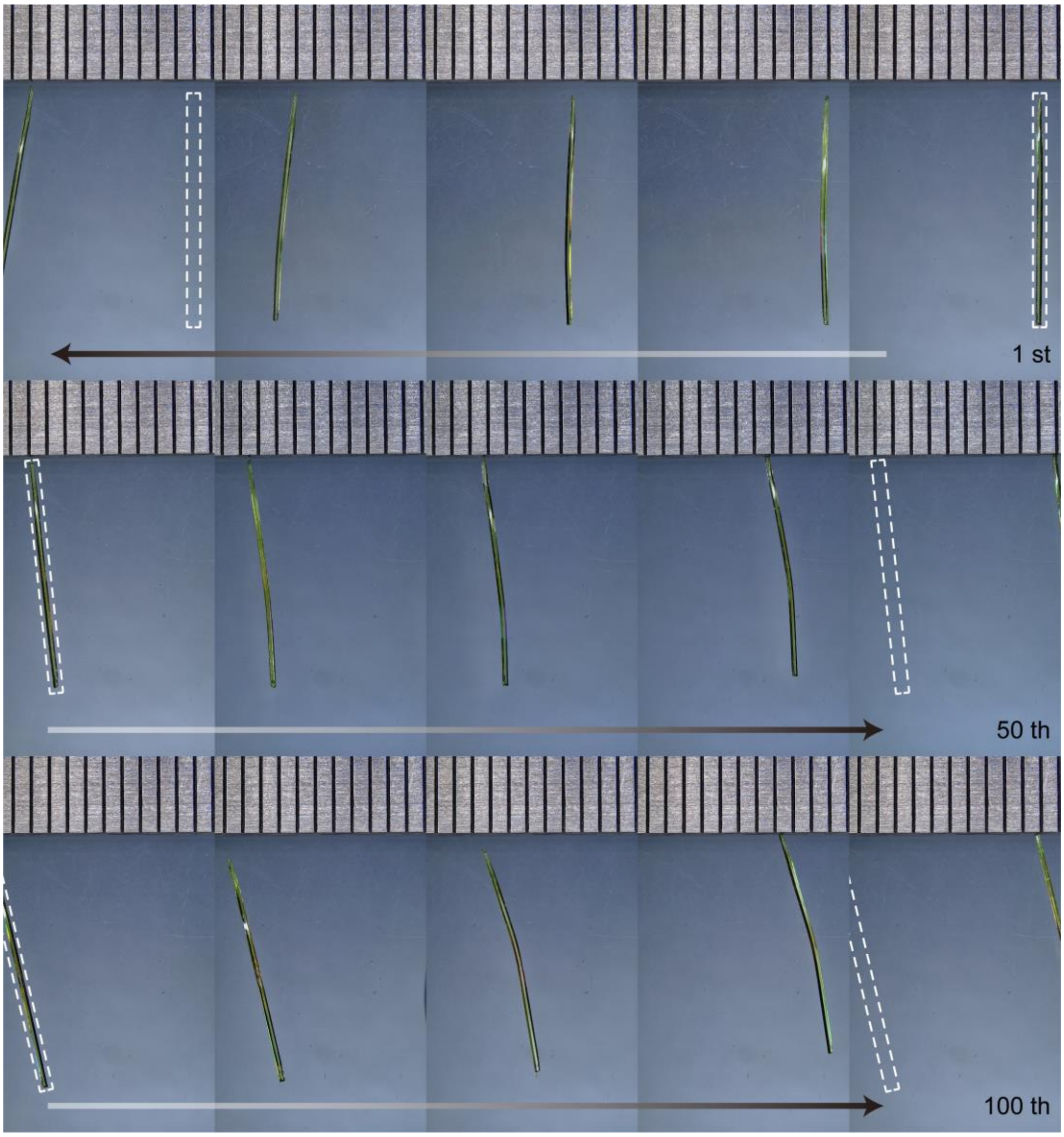

Figure S21. Microphotographs of back-and-forth rolling of an (*S*)-NTIEP single crystal exposed to UV radiation. The initial position of the single crystal was marked with a dashed box.

Supplementary Tables

Table S1. Crystal structure details for an (*S*)-NTIEP single crystal

	(*S*)-NTIEP
Formula	$C_{18}H_{18}N_2O_3$
Space Group	$P2_12_12_1$
Crystal System	Orthorhombic
a (Å)	5.23720(4)
b (Å)	11.50959(8)
c (Å)	25.25947(17)
α (deg)	90
β (deg)	90
γ (deg)	90
V (Å^3)	1522.591(19)
Z	4
R_1	0.0310
wR_2	0.0887
Temperature (K)	113
CCDC	2338790

Note: The hydrogen atoms attached to carbon atoms were placed at calculated positions, while the hydrogen atom attached to the nitrogen atom was located by residual electron density during the structure refinement.

Table S2. Crystal structures of (*S*)-NTIEP determined at different temperatures

Temperature	303 K	313 K	323 K	333 K	343 K	353 K
Formula	$C_{18}H_{18}N_2O_3$	$C_{18}H_{18}N_2O_3$	$C_{18}H_{18}N_2O_3$	$C_{18}H_{18}N_2O_3$	$C_{18}H_{18}N_2O_3$	$C_{18}H_{18}N_2O_3$
Space Group	$P2_12_12_1$	$P2_12_12_1$	$P2_12_12_1$	$P2_12_12_1$	$P2_12_12_1$	$P2_12_12_1$
Crystal System	orthorhombic	orthorhombic	orthorhombic	orthorhombic	orthorhombic	orthorhombic
a (Å)	5.45630(10)	5.47550(10)	5.49610(10)	5.51600(10)	5.54040(10)	5.56250(10)
b (Å)	11.46950(10)	11.4648(2)	11.45860(10)	11.4534(2)	11.4463(2)	11.4413(2)
c (Å)	25.1429(3)	25.1237(3)	25.1172(3)	25.1042(3)	25.0874(4)	25.0731(4)
α (deg)	90	90	90	90	90	90
β (deg)	90	90	90	90	90	90
γ (deg)	90	90	90	90	90	90
V (Å^3)	1573.47(4)	1577.15(8)	1581.82(4)	1586.01(4)	1590.97(5)	1595.71(5)
Z	4	4	4	4	4	4
R_1	0.0330	0.0336	0.0339	0.0363	0.0368	0.0373
wR_2	0.0977	0.1022	0.1028	0.1083	0.1081	0.1144
CCDC	2338794	2338795	2338797	2338798	2338800	2338801

363 K	373 K	383 K	393 K	403 K	413 K	303 K (R)
$C_{18}H_{18}N_2O_3$	$C_{18}H_{18}N_2O_3$	$C_{18}H_{18}N_2O_3$	$C_{18}H_{18}N_2O_3$	$C_{18}H_{18}N_2O_3$	$C_{18}H_{18}N_2O_3$	$C_{18}H_{18}N_2O_3$
$P2_12_12_1$	$P2_12_12_1$	$P2_12_12_1$	$P2_12_12_1$	$P2_12_12_1$	$P2_12_12_1$	$P2_12_12_1$
orthorhombic	orthorhombic	orthorhombic	orthorhombic	orthorhombic	orthorhombic	orthorhombic
5.58840(10)	5.61770(10)	5.6483(2)	5.67700(10)	5.7091(2)	5.7428(2)	5.46510(10)
11.4333(2)	11.4236(2)	11.4101(4)	11.4005(2)	11.3995(3)	11.3812(4)	11.46630(10)
25.0447(5)	25.0244(5)	25.0063(8)	24.9794(5)	24.9540(8)	24.9250(9)	25.1287(3)
90	90	90	90	90	90	90
90	90	90	90	90	90	90
90	90	90	90	90	90	90
1600.20(5)	1605.92(5)	1611.60(10)	1616.68(5)	1624.03(9)	1629.10(10)	1574.68(4)
4	4	4	4	4	4	4
0.0372	0.0387	0.0383	0.0389	0.0397	0.0394	0.0339
0.1179	0.1250	0.1232	0.1248	0.1295	0.1357	0.0998
2338802	2338804	2338805	2338806	2338807	2338808	2338809

Table S3. Thermal expansion coefficients reported for some organic crystals

Compound	Temperature Range (K)	TE coefficient(s)				Reference
		α_{X1} (10^{-6} K^{-1})	α_{X2} (10^{-6} K^{-1})	α_{X3} (10^{-6} K^{-1})	α_V (10^{-6} K^{-1})	
(*S*,*S*)-octa-3,5-diyn-2,7-diol	225-330	$156 < \alpha_a < 515$	$-32 < \alpha_b < -85$	$-48 < \alpha_c < -204$	47	[1]
MBDA (Form I)	148-298	24	43	70	146	[2]
MBDA (Form II)	148-298	-174	174	229	235	[2]
2-Butynoic acid (0D)	118-226	-	-	-	332	[3]
2-Butynoic acid (1D)	118-298	-	-	-	265	[3]
2,4-dinitroanisole (α)	100-293	13.7	26.6	136	176	[4]
2,4-dinitroanisole (β)	100-261	-31	-15	262	216	[4]
IMACET (Form I)	298-373	α_a=225.9	α_b=238.8	α_c=290.0	181.2	[5]
4-aminobenzonitrile	180-300	24.8	61.3	138.2	225.1	[6]
4-aminobenzonitrile	100-160	10.9	54.8	174.7	250	[6]
1,4-bis(1′-hydroxycyclopentyl)-1,3-butadiyne	100-340	-116	118	184	185	[7]
TI (Form II)	cooling	-17	28	145	161	[8]
TI (Form III)	cooling	-5	59	98	150	[8]
2-(4-bromophenyl)-4,5-dihydro-1H-imidazole (1N)	230-330	-175	-46	381	155	[9]
2-(4-bromophenyl)-4,5-dihydro-1H-imidazole (1N')	100-310	-189	151	255	217	[9]
BNDI-T	90-375	α_a=452	α_b=-16.8	α_c=-154	273	[10]
BNDI-M	90-375	α_a=70.1	α_b=-44.7	α_c=163	177	[10]
Olefin-I	190-290	-2	68	128	194	[11]
guanidinium nitrate	100-294	-5	-6.8	211.5	210.8	[12]
6-chloronicotinic acid	270-390	20.8	22.2	194.2	240.4	[13]
diazo-I	280-320	-65	64	211	210	[14]
(*S*)-NTIEP	303-413	-78	-69	465	323	This work
CBTA·BPE (Form I)	120-298	4	25	147	183	[15]
CBTA·BPE (Form II)	120-298	-4	18	136	158	[15]
nitromethane solvate of 18-crown-6	90-273	-129	144	286	311	[16]

2(4PAzP)·(4,6-diCl res)	260-290	-116	29	316	229	[17]
2(4PAzP)·(4,6-diBr res)	170-250	10	111	125	249	[17]
$C_{12}H_{24}O_6 \cdot 2CH_3NO_2$	180-273	-198	215	369	378	[18]
$C_{12}H_{24}O_6 \cdot 2CH_3CN$	180-273	-42	103	162.8	226	[18]
$C_{12}H_{24}O_6 \cdot 2CH_3I$	180-273	104	-1	153	256	[18]
2(4,6-diCl res)·2(4,4′-AP)	190-290	2	17	164	185	[19]
2(4,6-diBr res)·2(4,4′-AP)	190-290	-2	9	172	181	[19]
2(4,6-diI res)·2(4,4′-AP)	190-290	-4	19	178	196	[19]
ABN·2DMABN	100-300	24.3	90.9	105	222	[20]
IMD-HBC	100-360	-115	18	210	110	[21]
4-MIMD-HBC	100-350	-15	-12	92	67	[22]
RC·BPDI	190-290	-4	66	111	174	[23]
HQ·BPDI	190-290	-17	46	155	184	[23]
$PG_2 \cdot BPDI_3$	190-290	-5	66	94	155	[23]
$RV_2 \cdot BPDI_3$	190-290	-25	29	172	177	[23]

Supplementary Notes

1. **Excited-state intramolecular proton transfer (ESIPT) property:** The potential ESIPT property of (*S*)-NTIEP molecules in solution was indicated by fluorescence spectral analysis. Furthermore, we performed density functional theory (DFT) calculations to support the experimental result. All calculations were carried out with the Gaussian 16 software.[24] The ωb97XD functional[25] and def2-SVPD basis set were adopted for excited states geometry optimization and frequency calculations. The excited states were calculated with time-dependent DFT (TDDFT) method. The S_1 single point energy calculations were performed with the def2-TZVPD basis set. The SMD implicit solvation model[26] was used to account for the solvation effect (*n*-hexane and acetonitrile). Finally, the single point energy of each compound was added to the free energy correction terms calculated before to obtain the Gibbs free energy.

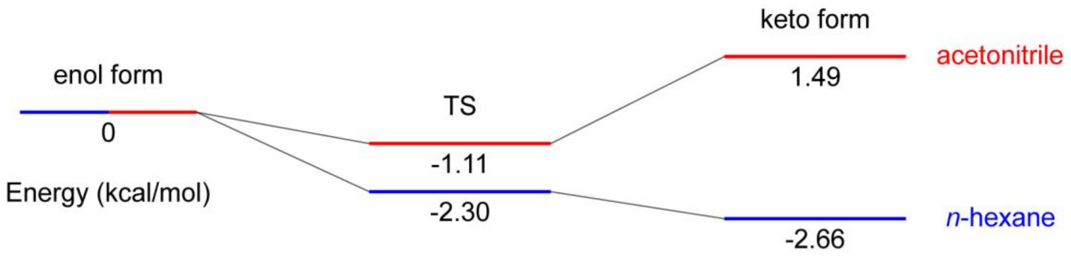

Figure S22. The calculated Gibbs free energy of (*S*)-NTIEP in the excited state solvated in *n*-hexane and acetonitrile.

The calculated Gibbs free energy in the excited state demonstrated a negligible energy barrier for proton transfer between the enol and keto tautomers, suggesting their facile interconversion. Notably, the calculations show that the Gibbs free energy of TS is lower than that of the enol form when the free energy correction is included, which is a normal phenomenon in DFT calculations.[27,28]

2. **Crystal structure analysis:** The (*S*)-NTIEP single crystal displays mechanical flexibility when subjected to applied force in different directions, which is attributed to its anisotropic crystal structure. Nowadays, understanding the mechanisms of elastic and plastic bending from the perspective of crystal structure and intermolecular interaction pioneered by C. Malla Reddy and Gautam R. Desiraju,[29] has shifted to experimental determination using micro-focus X-ray diffraction technique led by John C. McMurtrie and Jack K. Clegg.[30] Several universal models have been established to explain the mechanical properties of molecular crystals and have been widely used in most related studies. Therefore, we analyzed the origin of mechanical properties from the perspective of molecular packing and intermolecular interaction based on crystallographic data.

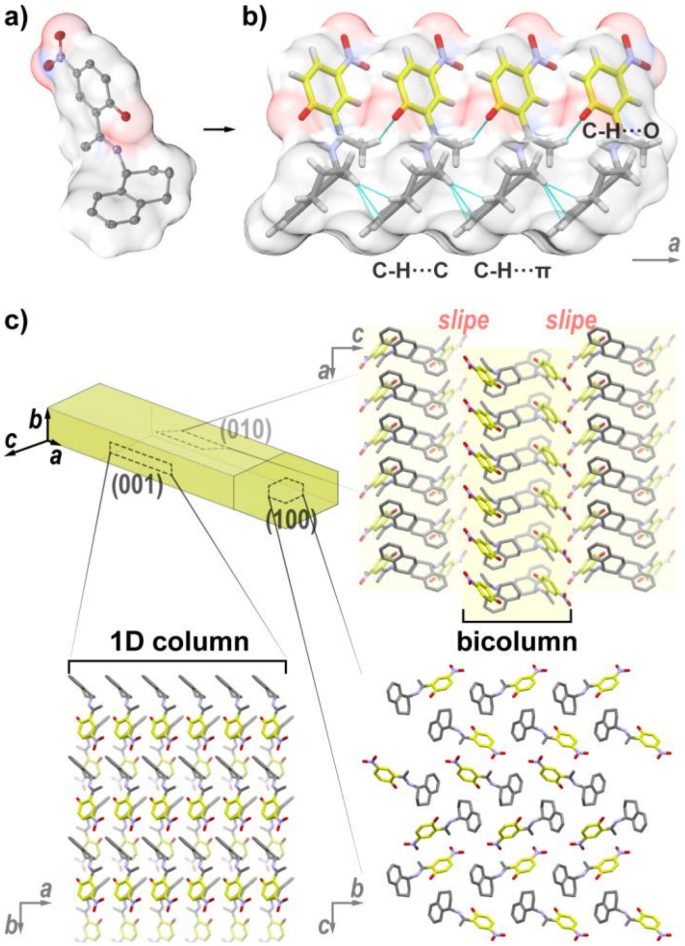

Figure S23. The crystal structure of (*S*)-NTIEP. (a) The asymmetric unit of (*S*)-NTIEP single crystal. (b) The one-dimensional chain along the crystallographic *a*-axis. (c) The crystal packing viewed along the [100], [010] and [001] directions.

The molecules form intramolecular N-H···O hydrogen bonds (with O···H distance of 1.747 Å) and exist in highly twisted conformation, which connect neighboring molecules via weak intermolecular C-H···O interactions (with O···H distance of 2.474 Å) as well as abundant C-H···C and C-H···π interactions to form one-dimensional chains which parallel to the crystallographic *a*-axis (the dominant growth direction). These chains assemble into two-dimensional layers that extend in the *ab*-plane and are further arranged staggered along the crystallographic *c*-axis into three-dimensional anisotropic packing structure. Besides, within the structure of (*S*)-NTIEP the slip planes are formed between bicolumns decorated with nitro groups on each side viewed along the crystallographic *b*-axis, which accords with slip-plane model of plastic bending when applied force along the [001] direction.

3. **Study of analogous compounds to (*S*)-NTIEP:** To assess whether similar thermal expansion occurs in other systems, we prepared several analogous compounds to (*S*)-NTIEP for comparative studies of the thermal expansion. Surprisingly, by replacing the nitro group in (*S*)-NTIEP molecule with -CF_3 and -CN as substituents (the respective compounds are denoted (*S*)-CF_3 and (*S*)-CN, respectively), we observed disparate thermal responses: the (*S*)-CF_3 crystal exhibited significant, albeit not outstanding, thermal expansion, while the (*S*)-CN crystal displayed normal thermal

expansion. Furthermore, we determined their single crystal structures, which provide further insights into their thermal expansion.

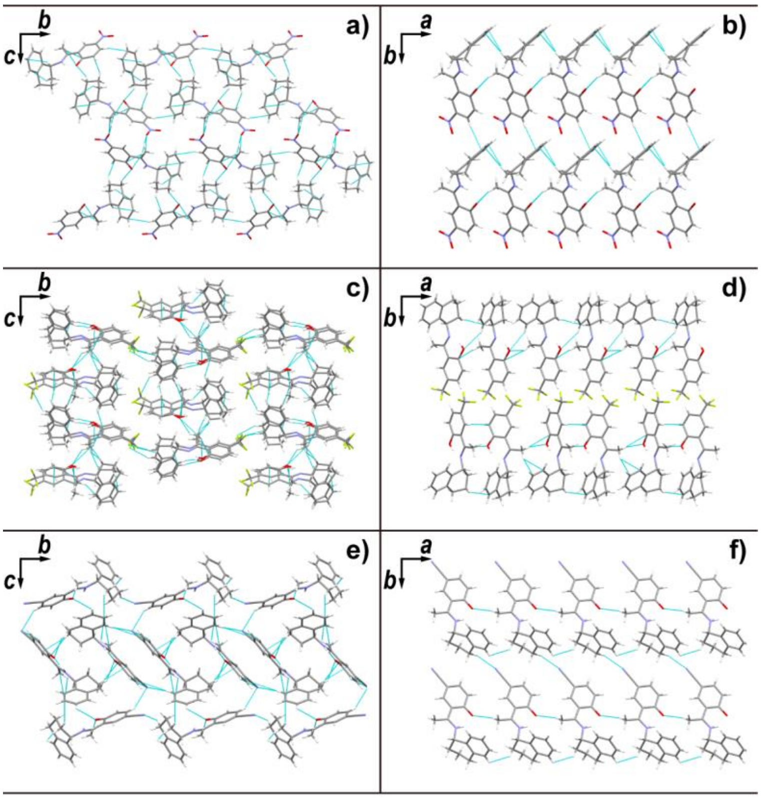

Figure S24. Comparison of crystal structures: the crystal packing of (a, b) (*S*)-NTIEP, (c, d) (*S*)-CF_3, and (e, f) (*S*)-CN, viewed along the crystallographic *a*-axis and *c*-axis.

Crystals of both (*S*)-CF_3 and (*S*)-CN exhibit needle-like morphologies, with the main growth direction along the crystallographic *a*-axis. Comparative analysis of their packing motifs revealed structural differences: in the crystal of (*S*)-CN, the hydrogen bonding along the [100] direction dominates the intermolecular interactions, the intermolecular spacing approaches 7 Å, and there are no significant C-H···π interactions. These features, at least qualitatively, do not appear to be favorable for molecular rearrangement during heating. In the crystal of (*S*)-CF_3, the intermolecular interactions and molecular packing are similar to those of (*S*)-NTIEP. Collectively, these experimental findings suggest that the relationships with structural signatures remain elusive. However, as we can conclude from our decade-long experience with similar phenomena, the colossal thermal expansion observed with this material is not an isolated phenomenon, and if taken together with other reported examples, could provide some guidance for future “design”.

Supplementary References

[1] D. Das, T. Jacobs, L. J. Barbour, *Nat. Mater.* **2010**, 9, 36-39.

[2] S. Bhattacharya, B. K. Saha, *CrystEngComm* **2014**, 16, 2340-2343.

[3] V. G. Saraswatula, S. Bhattacharya, B. K. Saha, *New J. Chem.* **2015**, 39, 3345-3348.

[4] H. Takahashi, R. Tamura, *CrystEngComm* **2015**, 17, 8888-8896.

[5] M. K. Panda, R. Centore, M. Causa, A. Tuzi, F. Borbone, P. Naumov, *Sci. Rep.* **2016**, 6, 29610.

[6] L. O. Alimi, D. P. van Heerden, P. Lama, V. J. Smith, L. J. Barbour, *Chem. Commun.* **2018**, 54, 6208-6211.

[7] D. Das, L. J. Barbour, *CrystEngComm* **2018**, 20, 5123-5126.

[8] A. Janiak, C. Esterhuysen, L. J. Barbour, *Chem. Commun.* **2018**, 54, 3727-3730.

[9] S. Dutta, P. Munshi, *J. Phys. Chem. C* **2020**, 124, 27413-27421.

[10] M. Dharmarwardana, B. M. Otten, M. M. Ghimire, B. S. Arimilli, C. M. Williams, S. Boateng, Z. Lu, G. T. McCandless, J. J. Gassensmith, M. A. Omary, *Proc. Natl. Acad. Sci. USA* **2021**, 118, e2106572118.

[11] X. Ding, E. Zahid, D. K. Unruh, K. M. Hutchins, *IUCrJ* **2022**, 9, 31-42.

[12] D. P. Karothu, R. Ferreira, G. Dushaq, E. Ahmed, L. Catalano, J. M. Halabi, Z. Alhaddad, I. Tahir, L. Li, S. Mohamed, M. Rasras, P. Naumov, *Nat. Commun.* **2022**, 13, 2823.

[13] J. Lin, Z. Guo, K. Zhang, P. Zhao, S. Wu, J. Xu, J. Gong, Y. Bao, *Adv. Funct. Mater.* **2022**, 2203004.

[14] X. Ding, D. K. Unruh, L. Ma, E. J. van Aalst, E. W. Reinheimer, B. J. Wylie, K. M. Hutchins, *Angew. Chem. Int. Ed.* **2023**, 62, e202306198.

[15] S. Bhattacharya, B. K. Saha, *Cryst. Growth Des.* **2013**, 13, 3299-3302.

[16] E. R. Engel, V. J. Smith, C. X. Bezuidenhout, L. J. Barbour, *Chem. Commun.* **2014**, 50, 4238-4241.

[17] K. M. Hutchins, R. H. Groeneman, E. W. Reinheimer, D. C. Swenson, L. R. MacGillivray, *Chem. Sci.* **2015**, 6, 4717-4722.

[18] E. R. Engel, V. J. Smith, C. X. Bezuidenhout, L. J. Barbour, *Chem. Mater.* **2016**, 28, 5073-5079.

[19] K. M. Hutchins, K. A. Kummer, R. H. Groeneman, E. W. Reinheimer, M. A. Sinnwell, D. C. Swenson, L. R. MacGillivray, *CrystEngComm* **2016**, 18, 8354-8357.

[20] L. O. Alimi, P. Lama, V. J. Smith, L. J. Barbour, *CrystEngComm* **2018**, 20, 631-635.

[21] B. Dwivedi, A. Shrivastava, L. Negi, D. Das, *Cryst. Growth Des.* **2019**, 19, 2519-2524.

[22] S. Chuskit, T. Sethi, P. Harsha, D. Das, *Cryst. Growth Des.* **2023**, 23, 1336-1342.

[23] N. Juneja, D. K. Unruh, G. C. George, III, K. M. Hutchins, *Cryst. Growth Des.* **2023**, 23, 524-531.

[24] M. J. Frisch, G. W. Trucks, H. B. Schlegel, G. E. Scuseria, M. A. Robb, J. R. Cheeseman, G. Scalmani, V. Barone, G. A. Petersson, H. Nakatsuji, X. Li, M. Caricato, A. V. Marenich, J. Bloino, B. G. Janesko, R. Gomperts, B. Mennucci, H. P. Hratchian, J. V. Ortiz, A. F. Izmaylov, J. L. Sonnenberg, Williams, F. Ding, F. Lipparini, F. Egidi, J. Goings, B. Peng, A. Petrone, T. Henderson, D. Ranasinghe, V. G. Zakrzewski, J. Gao, N. Rega, G. Zheng, W. Liang, M. Hada, M. Ehara, K. Toyota, R. Fukuda, J. Hasegawa, M. Ishida, T. Nakajima, Y. Honda, O. Kitao, H. Nakai, T. Vreven, K. Throssell, J. A. Montgomery Jr., J. E. Peralta, F. Ogliaro, M. J. Bearpark, J. J. Heyd, E. N. Brothers, K. N. Kudin, V. N. Staroverov, T. A. Keith, R. Kobayashi, J. Normand, K. Raghavachari, A. P. Rendell, J. C. Burant, S. S. Iyengar, J. Tomasi, M. Cossi, J. M. Millam, M. Klene, C. Adamo, R. Cammi, J. W. Ochterski, R. L. Martin, K. Morokuma, O. Farkas, J. B. Foresman, D. J. Fox, *Gaussian 16 Rev. C.01*, Wallingford, CT, **2016**.

[25] J.-D. Chai, M. Head-Gordon, *Phys. Chem. Chem. Phys.* **2008**, 10, 6615-6620.

[26] A. V. Marenich, C. J. Cramer, D. G. Truhlar, *J. Phys. Chem. B* **2009**, 113, 6378-6396.

[27] Y.-X. Zhao, X.-N. Wu, J.-B. Ma, S.-G. He, X.-L. Ding, *J. Phys. Chem. C* **2010**, 114, 12271-12279.

[28] L. Wang, J.-J. Wu, C.-C. Yan, W.-Y. Yang, Z.-L. Che, X.-Y. Xia, X.-D. Wang, L.-S. Liao, *Chin. Chem. Lett.* **2024**, **35**, 109365.

[29] S. Saha, M. K. Mishra, C. M. Reddy, G. R. Desiraju, *Acc. Chem. Res.* **2018**, 51, 2957-2967.

[30] A. J. Thompson, J. A. Powell, J. N. Melville, J. C. McMurtrie, J. K. Clegg, *Small* **2023**, 19, 2207431.
